# Exploring partially shared valuation-related neural responses between abstract desirability and food incentive evaluation

**DOI:** 10.3389/fnhum.2026.1909419

**Published:** 2026-09-30

**Authors:** Renkai Chen, Fufang Wang

**Affiliations:** 1 Taizhou College, Taizhou, Zhejiang, China; 2 Research Institute of Foreign Languages, Bejing Foreign Studies University, Beijing, China

**Keywords:** abstract concepts, desire, fMRI, food incentive value, hunger, motivational valuation

## Abstract

Motivational evaluation can generalize across diverse goals, while the incentive value of biologically relevant stimuli such as food is dynamically modulated by physiological need. The present study examined whether cross-category desirability evaluation under a non-deprived baseline condition and food incentive evaluation under experimentally manipulated Hunger and Satiety states show partially similar valuation-related neural responses within the same individuals. Participants underwent functional magnetic resonance imaging (fMRI) while completing two complementary experiments: an abstract desirability judgment task involving objects, events, and persons, and a food evaluation task conducted under Hunger and Satiety states. In the DESIRE experiment, neural responses associated with desirable versus undesirable judgments were examined across three semantic categories to identify candidate neural patterns related to abstract desirability evaluation. In the HUNGER experiment, analyses examined both baseline food incentive value and hunger-related increases in food desirability. Exploratory whole-brain analyses identified activation patterns involving medial frontal and cingulate valuation-related regions, including the medial prefrontal cortex (mPFC), anterior cingulate cortex (ACC), and medial orbitofrontal cortex (mOFC), during abstract desirability evaluation. Exploratory maps in baseline food incentive-value evaluation analysis also implicated medial frontal and cingulate valuation-related regions. In addition, food items whose desirability increased under hunger showed greater activation than rating-stable food items in the left inferior orbitofrontal cortex, together with visual, cerebellar, and sensorimotor regions. However, formal conjunction-null analyses, ROI-based convergence analyses, whole-brain overlap analyses, and permutation-based tests did not provide evidence for a statistically robust shared neural representation across abstract desirability and food valuation. These findings suggest that cross-category desirability evaluation and homeostatically modulated food incentive evaluation may involve partially overlapping valuation-related processes while remaining sensitive to semantic context and physiological state. The results provide preliminary support for the possibility that abstract motivational cognition may partially engage valuation-related processes that are also involved in physiological incentive evaluation.

## 1 Introduction

Understanding how abstract concepts are represented and processed in the human brain remains a major challenge in cognitive neuroscience. Unlike concrete concepts, which often engage modality-specific sensory and motor systems, abstract concepts rely on distributed neural networks related to affective, motivational, and contextual processing (Hauk et al., 2004; Barsalou and Wiemer-Hastings, 2005; Barsalou, 2008; Pecher et al., 2011; Barsalou et al., 2018). Yet, treating abstract concepts as a homogeneous category may risk obscuring significant differences in their semantic content and cognitive function. Recent theories emphasize that abstract concepts are highly heterogeneous, with their neural representations relying heavily on the specific types of information they encode, including social interaction, emotion, value-related knowledge, or internal bodily states (Barsalou and Wiemer-Hastings, 2005; Kousta et al., 2011; Vigliocco et al., 2014; Connell et al., 2018; Fingerhut and Prinz, 2018; Borghi et al., 2022).

Among different classes of abstract concepts, motivational concepts are important in that they are closely linked to internal states, value computation, and goal-directed behavior. Rather than being defined primarily by perceptual features, motivational concepts encode information about what is desirable, relevant, and worth pursuing (Berridge, 2004; Bissonette and Roesch, 2016; Yee, 2024). Desire represents a useful case of such abstract motivational cognition. Although desire does not correspond to a single external object or sensory modality, it reflects a motivational orientation toward obtaining, experiencing, or becoming something desirable (Papies and Barsalou, 2015; Kiverstein et al., 2025). Neuroimaging research has implicated medial frontal and cingulate regions, including the anterior cingulate cortex (ACC), medial prefrontal cortex (mPFC), and orbitofrontal cortex (OFC), in subjective value computation, motivational relevance, and integration of internal states with anticipated outcomes (Cardinal et al., 2002; Kawabata and Zeki, 2008; Kim, 2013; Weinstein, 2023; Yee, 2024). Within this framework, it’s important to distinguish between wanting and liking. Wanting refers to motivational attraction, incentive salience, and the drive to obtain a reward, whereas liking reflects the hedonic pleasure associated with reward consumption (Berridge and Kringelbach, 2015; Morales and Berridge, 2020; Bosulu et al., 2022; Skov, 2023). Because desire primarily concerns motivational orientation rather than immediate sensory pleasure, the present study focuses on motivational desirability (“wanting”), while recognizing that wanting and liking represent related but dissociable components of reward processing.

Importantly, desire can be directed toward semantically very different targets, including objects, events, and persons. Examining desirability across such heterogeneous targets provides a way to identify candidate valuation-related responses that generalize across semantic domains and are therefore less likely to reflect category-specific semantic content alone (Fairhall and Caramazza, 2013; Berridge, 2023). In the present study, “abstract desirability” does not refer to the perceptual concreteness of the stimuli themselves, nor is it defined simply by the number of semantic categories being evaluated. Rather, the DESIRE experiment was designed to characterize desirability as a motivational dimension that can generalize across semantically heterogeneous targets. Objects, events, and persons were sampled as distinct target domains so that candidate desirability-related responses shared across them would be less likely to reflect category-specific semantic content alone. Importantly, these judgments were assessed under a non-deprived baseline condition and were not experimentally coupled to an acute homeostatic need manipulation. Thus, “abstract desirability” is used here to refer to cross-category motivational evaluation that is not experimentally driven by an acute homeostatic state, rather than to perceptually abstract stimuli (Kawabata and Zeki, 2008).

Hunger provides a biologically grounded reference point for investigating physiological incentive valuation. As an interoceptive state, hunger arises from signals originating within the body and serves to maintain physiological homeostasis. It can systematically increase the incentive value of food, leading to measurable changes in both behavior and neural activity (Burnett et al., 2016; Stevenson et al., 2023). Neuroimaging research has shown that hunger and food-related evaluation are associated with activation in valuation-related regions, including the OFC, ACC, insula, and medial frontal cortex (Tataranni et al., 1999; Siep et al., 2009; Brooks et al., 2013). Thus, food motivation provides an experimentally tractable model for investigating how internal physiological states shape incentive valuation and how bodily needs influence the motivational relevance of external stimuli. In the present study, the physiological character of the HUNGER experiment therefore derives not from food stimuli *per se*, but from the experimental coupling of food incentive value to acute bodily-state manipulation. The same food items were evaluated under experimentally induced Hunger and Satiety states, allowing food desirability to be examined as a function of changes in homeostatic need.

Accordingly, the DESIRE and HUNGER experiments differed along three theoretically relevant dimensions. First, they differed in physiological-state dependency. The DESIRE experiment assessed desirability under a non-deprived baseline condition without experimentally manipulating an acute bodily need, whereas the HUNGER experiment explicitly manipulated physiological state through food deprivation and subsequent satiety (Morton et al., 2014). Second, they differed in homeostatic relevance. Desirability judgments toward objects, events, and persons were not directly tied to restoration of an experimentally induced physiological deficit, whereas food evaluation was embedded in a homeostatic context in which changes in hunger and satiety altered the motivational significance of food (Arana et al., 2003; Morton et al., 2014; Campos et al., 2022). Third, they differed in semantic-domain breadth. The DESIRE experiment sampled motivational evaluation across three semantically distinct target domains, whereas the HUNGER experiment examined valuation within the single broad domain of food. This third distinction served as an experimental means of testing cross-category generalization and was not, by itself, treated as the defining criterion of abstractness (Kawabata and Zeki, 2008; Bartra et al., 2013). Importantly, food evaluation itself also engages general subjective valuation processes. The distinction between the two experiments is therefore not a categorical opposition between “abstract” and “physiological” motivation. Rather, the comparison concerns whether cross-category desirability evaluation assessed under a non-deprived baseline condition and food incentive evaluation directly modulated by homeostatic state recruit partially similar valuation-related neural processes. The presence of a shared evaluative component is precisely what makes this comparison theoretically informative.

Against this background, comparing the two experiments is also relevant to embodied cognition and primary metaphor accounts. In Grady’s formulation, primary metaphors arise from recurrent experiential correlations between comparatively basic bodily or sensory source experiences and subjective target experiences such as judgments, evaluations, or motivational responses. DESIRE IS HUNGER is theoretically distinctive in this respect because its source domain is an interoceptive and homeostatically relevant bodily state rather than an external sensory or spatial experience (Grady, 1997; Lakoff and Johnson, 1999). From this perspective, partial correspondence between generalized desirability and homeostatically modulated food valuation would be compatible with the possibility that higher-level motivational evaluation draws partly on valuation-related processes also engaged by bodily motivational states, without implying identical neural representations.

The present study employed functional magnetic resonance imaging (fMRI) to compare two complementary forms of motivational evaluation within the same individuals: cross-category desirability evaluation assessed under a non-deprived baseline condition and food incentive evaluation assessed under experimentally manipulated Hunger and Satiety states. Rather than treating these as mutually exclusive categories of “abstract” and “physiological” motivation, the study examined whether valuation-related neural responses generalize across motivational contexts that differ in physiological-state dependency, homeostatic relevance, and semantic-domain breadth. The DESIRE experiment examined desirability judgments across three semantic categories (objects, events, and persons) to identify candidate category-independent activation patterns associated with abstract desirability evaluation. The HUNGER experiment examined food evaluation under Hunger and Satiety states to characterize both baseline food incentive value and hunger-related increases in food desirability.

Using these complementary experiments, we addressed three related questions. First, we examined whether desirability-related response generalized across semantically heterogeneous target domains in the DESIRE experiment, identifying candidate valuation-related activity that was less likely to reflect category-specific semantic content alone. Second, we tested whether baseline food incentive-value responses show partially similar activation patterns with abstract desirability evaluation. Third, we investigated whether food stimuli whose desirability increased under hunger engaged additional neural systems related to perceptual salience and action-related processing. Taken together, these analyses were designed to examine the extent and limits of cross-domain convergence between cross-category desirability evaluation and homeostatically modulated food incentive evaluation, while considering the possibility that motivational evaluation may involve partially shared but heterogeneous neural processes rather than a single domain-general neural code.

## 2 Materials and methods

The study employed a within-subject functional magnetic resonance imaging (fMRI) design to examine neural responses associated with abstract desirability evaluation and physiological food motivation. Participants completed two complementary experiments: the DESIRE experiment, which assessed desirability judgments across multiple semantic categories, and the HUNGER experiment, which examined food incentive valuation under different physiological states. The following sections describe participant characteristics, experimental procedures, stimulus construction, imaging acquisition, and statistical analyses.

### 2.1 Participants

Ten healthy right-handed adults (5 males and 5 females; age range = 18–29 years, mean ± SD = 21.2 ± 3.6 years; body mass index (BMI) range = 17.3–32.6 kg/m^2^, mean ± SD = 22.1 ± 4.4 kg/m^2^) participated in the study.

All participants reported normal or corrected-to-normal vision and no history of neurological or psychiatric disorders, substance abuse, metabolic disease, or current dieting behavior. Participants provided written informed consent before participation. The study protocol was approved by the Ethics Committee of East China Normal University and was conducted in accordance with relevant ethical guidelines.

### 2.2 Experimental overview

The study employed a within-subject fMRI design consisting of two complementary experiments designed to examine motivational evaluation across different semantic and physiological contexts.

The DESIRE experiment examined desirability evaluation toward semantically diverse targets, including objects, events, and persons. This experiment was designed to identify neural responses associated with abstract motivational evaluation across different target domains. The HUNGER experiment examined food-related incentive valuation under two physiological states, Hunger and Satiety. This experiment was designed to characterize both baseline food incentive value and state-dependent changes in food desirability associated with hunger.

Together, these two experiments enabled within-subject comparison between cross-category desirability evaluation and food incentive valuation under manipulated physiological states, providing a framework for examining potential convergence and divergence in valuation-related neural processes. The overall experimental design and task procedures are illustrated in Figure 1.

**Figure 1:**
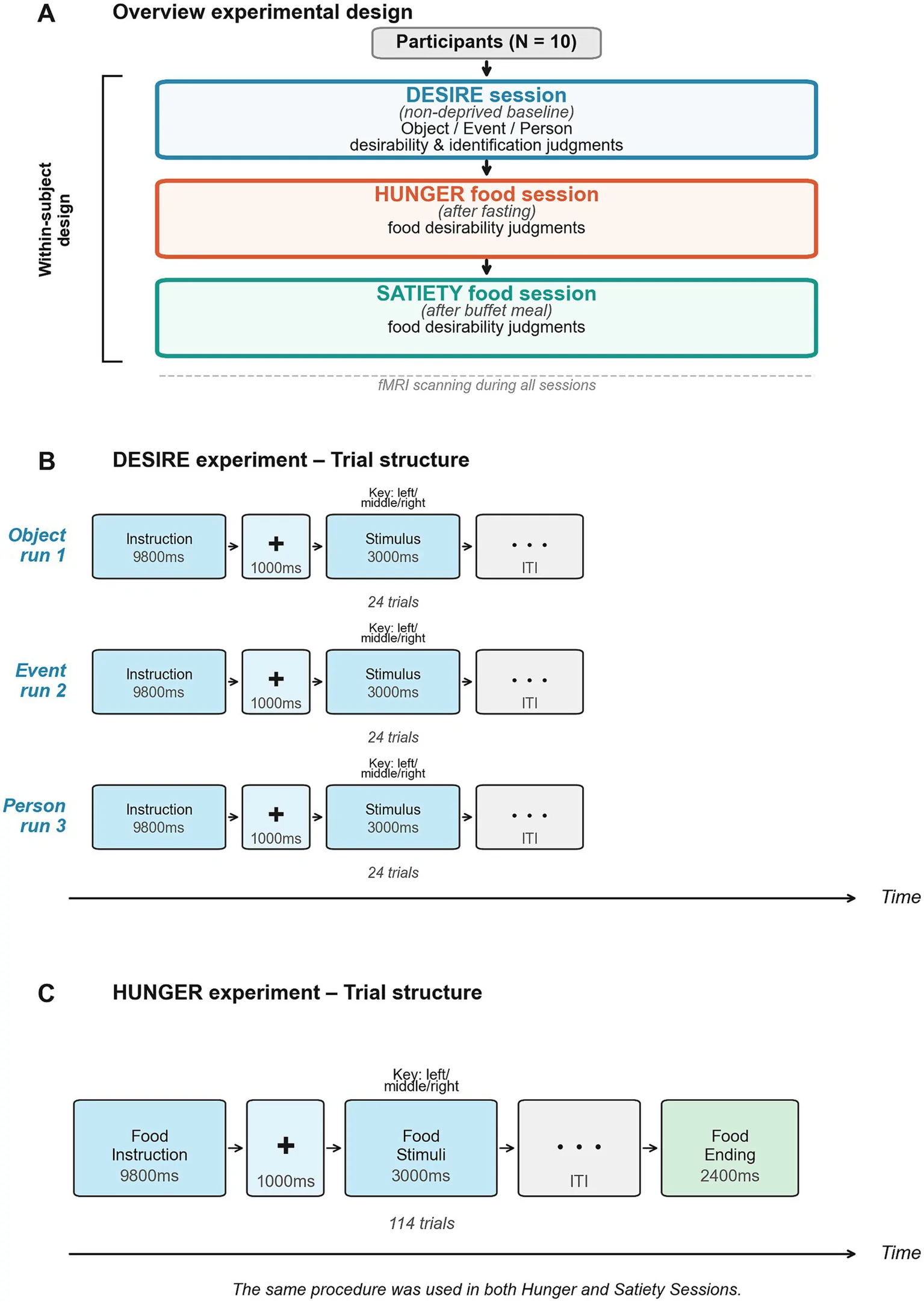
Experimental design and task procedures. **(A)** Overview of the within-subject experimental design. Participants completed an abstract desirability judgment session under a non-deprived baseline state and food desirability judgment sessions under Hunger and Satiety conditions. **(B)** Trial structure of the abstract desirability judgment task. Object, event, and person stimuli were presented in separate runs, and participants rated each stimulus according to subjective desirability. **(C)** Trial structure of the food desirability judgment task. Food stimuli were rated according to subjective desirability. Identical event-related procedures were used in both Hunger and Satiety Sessions.

### 2.3 Stimuli

Custom image sets were developed for the two experiments to examine motivational desirability and food-related incentive value. Stimulus selection procedures were designed to emphasize subjective motivational desirability (“wanting”) rather than hedonic liking, although liking responses were not independently collected in the present study.

For the DESIRE experiment, an initial set of 1,245 images was selected across three semantic categories: objects, events, and persons. The stimuli were selected to cover a broad range of desirability values while minimizing extreme emotional content and category-related biases. Before fMRI scanning, an independent behavioral validation study was conducted with 41 healthy participants who did not participate in the neuroimaging experiment. Participants rated each image using a 10-point desirability scale (1 = least desirable; 10 = most desirable) after receiving instructions distinguishing motivational wanting/desirability from hedonic liking/pleasure. Based on mean ratings and variability criteria, 24 images from each desirability level (*desirable, indifferent,* and *undesirable*) were selected within each category, resulting in 216 stimuli for the fMRI experiment.

For the HUNGER experiment, an initial set of 250 familiar food images was selected to cover a broad range of potential food incentive values while minimizing differences in visual complexity. These images were preliminarily rated by an independent sample (*n* = 4) using the same desirability scale. Because food preferences are highly individualized, these preliminary ratings were used only for initial stimulus selection and balancing of the stimulus set. The food categories used in neuroimaging analyses were subsequently determined based on each participant’s own desirability ratings collected during the Hunger and Satiety Sessions. Based on the preliminary ratings, 38 images from each desirability category (*desirable, indifferent,* and *undesirable*) were selected, resulting in 114 food stimuli.

All stimuli were standardized in size (640 × 480 pixels) and resolution (100 DPI) and presented centrally against a neutral background. Detailed stimulus validation procedures and selection criteria are provided in Supplementary methods S1.

### 2.4 Procedure

Both DESIRE and HUNGER experiments employed an event-related design. Each trial began with a fixation cross (1,000 ms), followed by stimulus presentation (3,000 ms), during which participants indicated their subjective desirability judgment using a three-button response. Responses were made with the right hand and classified as desirable, indifferent, or undesirable. Trials were presented in randomized order within each run. The experimental design and task procedures are illustrated in Figure 1.

The DESIRE session consisted of desirability judgment runs and identification-based judgment runs for each stimulus category (objects, events, and persons). During desirability judgment runs, participants evaluated how much they wanted the depicted target, including wanting to obtain the object, participate in the event, or become the person represented in the image. During identification-based judgment runs, participants performed category-specific non-motivational judgments using the same stimuli, including ownership judgments for objects, experience judgments for events, and gender identification judgments for persons. Each run contained 72 trials and used identical stimulus timing parameters. The primary analyses focused on desirability judgment runs because they directly assessed motivational evaluation. Identification-based runs were included as an additional non-motivational comparison condition. Although they were not included in the primary analyses because the main objective was to characterize desirability-related valuation processes, desirability-versus-identification comparisons were conducted as supplementary control analyses.

Before the scanning session, participants received standardized instructions distinguishing motivational wanting from hedonic liking. They were informed that wanting refers to the motivation or desire to obtain, experience, or pursue an outcome, whereas liking refers to the pleasure associated with experiencing or consuming that outcome. Examples were provided to illustrate situations in which wanting and liking may differ, and this distinction was emphasized throughout the desirability judgment tasks.

The DESIRE session was conducted under a non-deprived baseline state. Participants were instructed to maintain their habitual eating routine before scanning and were scheduled approximately 2–3 h after a regular meal. Before scanning, participants reported their subjective fullness state using a 10-point visual analogue scale (VAS; 1 = extremely hungry, 10 = extremely full). These ratings were collected to characterize participants’ physiological state during the DESIRE session.

The HUNGER experiment consisted of two fMRI sessions conducted under different physiological states: Hunger and Satiety. The same event-related procedure was used in both sessions, during which participants evaluated how much they wanted to consume each food item using the same three-level desirability response format. The wanting–liking distinction was briefly reiterated before the HUNGER Session to ensure consistent interpretation of food desirability judgments.

To induce the Hunger state, participants were instructed to abstain from all food and caloric beverages for approximately 18 h before scanning, with water permitted. Before scanning, participants reported their subjective hunger/fullness state using the same VAS scale to characterize the effect of food deprivation.

To induce the Satiety state, participants consumed a buffet-style meal immediately before scanning. The buffet procedure provided a variety of foods to promote fullness while reducing sensory-specific satiety associated with consuming a single food type. Scanning began approximately 30 min after completion of the meal, and subjective fullness ratings were collected before scanning to characterize the satiated state. The order of Hunger and Satiety Sessions was counterbalanced across participants.

### 2.5 Imaging acquisition and preprocessing

MRI data were acquired at the Shanghai Key Laboratory of Magnetic Resonance (East China Normal University) using a 3 T Siemens Prisma scanner (Siemens, Germany) equipped with a 20-channel head coil.

High-resolution T1-weighted anatomical images were acquired using a three-dimensional spoiled gradient echo (SPGR) sequence (TR = 2,530 ms; TE = 2.34 ms; flip angle = 7°; FoV = 256 mm; voxel size = 1 × 1 × 1 mm^3^; 192 sagittal slices). These anatomical images were used for co-registration and spatial normalization.

Functional images were acquired using a T2*-weighted gradient-echo echo-planar imaging (EPI) sequence sensitive to BOLD contrast (TR = 2,450 ms; TE = 30 ms; flip angle = 81°; FoV = 192 mm; voxel size = 3 × 3 × 3 mm^3^; 40 axial slices acquired in interleaved order parallel to the AC–PC line; no slice gap). Each DESIRE run consisted of 122 volumes, and each HUNGER run consisted of 192 volumes.

Image preprocessing was performed using SPM8 (Wellcome Trust Centre for Neuroimaging, London, UK). The first four functional volumes of each run were discarded to allow for magnetization stabilization; this procedure was applied uniformly across participants and was unrelated to motion censoring or artifact removal. The remaining images were corrected for slice timing and realigned using a six-parameter rigid-body transformation. Functional images were then co-registered to individual T1-weighted anatomical images, normalized to the Montreal Neurological Institute (MNI-152) standard space, and spatially smoothed using a 5-mm full-width-at-half-maximum (FWHM) Gaussian kernel. A high-pass filter with a cutoff of 128 s was applied to remove low-frequency drift.

Motion parameters from realignment (three translations and three rotations) were included as nuisance regressors in the first-level GLM. No additional motion-outlier regressors or volume censoring procedures were included. No participant exceeded the predefined motion criteria of 3 mm translation or 3° rotation.

### 2.6 Statistical analysis

Neuroimaging analyses were conducted within the general linear model (GLM) framework. The exploratory whole-brain analyses reported in the primary Results sections were originally performed using SPM8. To further characterize the robustness and interpretability of the observed patterns, additional robustness analyses were conducted using the same dataset within SPM12, including ROI-based analyses, formal conjunction-null analyses, trial-wise parametric modeling, spatial overlap analyses, permutation-based robustness tests, and identification-control analyses. These analyses were not intended to increase statistical power, but rather to characterize the reliability and specificity of exploratory activation patterns. SPM12-based analyses used the same first-level contrast images and were performed to provide independent robustness evaluations rather than to replace the original exploratory analyses.

Statistical thresholds and reporting criteria were specified separately for exploratory whole-brain mapping and robustness analyses. Different exploratory thresholds were retained because these analyses were originally designed as hypothesis-generating investigations rather than confirmatory tests; none of these uncorrected thresholds were interpreted as confirmatory evidence. Whole-brain results were reported with voxel-wise thresholds, cluster-extent thresholds, cluster sizes, peak coordinates, and statistical values in Montreal Neurological Institute (MNI) space. Given the modest sample size, exploratory whole-brain analyses were interpreted cautiously, whereas ROI-based analyses incorporated multiple-comparison correction and standardized effect-size estimation with confidence intervals.

#### 2.6.1 First-level and second-level GLM analyses

For each participant, functional responses were modeled using event-related GLMs. Original first-level and second-level analyses were performed using SPM8, whereas additional robustness analyses used SPM12 based on the same first-level contrast structure. Stimulus onset times were convolved with the canonical hemodynamic response function. Six rigid-body motion parameters (three translations and three rotations) were included as nuisance regressors. No additional motion-outlier regressors or volume censoring procedures were applied. The first four functional volumes of each run were discarded to allow for magnetization equilibrium; this procedure was applied uniformly across participants and did not represent motion-based censoring.

For the DESIRE experiment, desirability judgment and identification judgment runs were modeled separately. In desirability runs, trials were modeled according to stimulus category (object, event, and person) and response group (desirable, indifferent, and undesirable), resulting in nine task regressors (3 stimulus categories × 3 response categories). Indifferent trials were modeled but were not included in the primary desirable-versus-undesirable contrasts. The primary contrast focused on the two extremes of the desirability continuum because the goal was to identify neural responses associated with motivational preference rather than to estimate a parametric linear value function. Identification runs were modeled separately as a non-motivational comparison condition. Additional identification-control analyses comparing desirability and identification judgments were conducted using predefined whole-brain and ROI-level contrasts and are reported in Supplementary Results SR2 and Supplementary Table S8.

For the HUNGER experiment, Hunger and Satiety Sessions were modeled separately to account for distinct physiological states. Within each session, food trials were modeled according to participants’ desirability responses (desirable, indifferent, and undesirable). Thus, each Hunger or Satiety Session GLM included three task regressors corresponding to desirable, indifferent, and undesirable food trials. Categorical food-value effects were assessed using desirable versus undesirable contrasts, whereas continuous food-value encoding was examined using trial-wise desirability ratings as parametric modulators (see Section 2.6.3).

Individual contrast images were entered into second-level random-effects analyses to estimate group-level effects. For the DESIRE experiment, category-specific desirability contrasts were calculated as *OT_desirable_ > OT_undesirable_, ET_desirable_ > ET_undesirable_*, and *PT_desirable_ > PT_undesirable_*. A combined abstract desirability contrast (*Desire_Combined*) was generated by averaging the three category-specific contrasts. For the HUNGER experiment, categorical food-value effects were assessed using *Food_desirable_ > Food_undesirable_* contrasts across physiological states.

#### 2.6.2 Whole-brain analyses of desirability and food-value responses

(1) Neural correlates of abstract desirability evaluation

To identify candidate neural regions associated with abstract desirability evaluation, an exploratory whole-brain conjunction analysis was conducted across OT, ET, and PT desirability contrasts. This exploratory conjunction analysis was designed to identify spatially overlapping candidate regions showing consistent desirability-related responses across the three semantic domains. The resulting activation patterns were interpreted as candidate neural correlates of desirability evaluation across semantic categories. In addition, a separate conjunction-null analysis was conducted as part of the robustness analyses described below.

(2) Neural correlates of baseline food incentive value

To characterize neural responses associated with baseline food incentive value, desirable food trials were contrasted with undesirable food trials across Hunger and Satiety Sessions. The Hunger and Satiety contrast images were entered as separate first-level contrast images into a second-level model estimating the average food-value effect across physiological states. This contrast was intended to identify regions associated with food motivational value independent of transient hunger-related changes. Whole-brain statistical maps were generated to identify regions associated with categorical food-value responses.

(3) Neural responses associated with hunger-related increases in food desirability

To investigate neural responses associated with hunger-related changes in food desirability, food stimuli were classified using participant-specific rating changes between Hunger and Satiety Sessions. Ratings were coded as: desirable = 1, indifferent = 2, and undesirable = 3; therefore, lower numerical values indicated greater subjective desirability. Food items were classified into three categories based on the comparison of ratings in Hunger and Satiety Sessions: Hunger Foods (Rating*_Hunger_* < Rating*_Satiety_*), indicating increased desirability during the Hunger Session; Neutral Foods (Rating*_Hunger_* = Rating*_Satiety_*), indicating stable desirability across physiological states; and Irregular Foods (Rating*_Hunger_* > Rating*_Satiety_*), indicating an opposite transition. Missing or invalid responses were excluded from classification and subsequent analyses. In our analysis, the primary hunger-related contrast compared HungerFood > NeutralFood within the Hunger Session.

Because this classification was based on participant-specific changes in desirability ratings across physiological states, this contrast was interpreted as reflecting neural responses associated with hunger-related changes in food desirability. It may also contain contributions from differences in current desirability levels during the Hunger Session; therefore, interpretation emphasized cautious characterization rather than a purely state-specific valuation mechanism.

#### 2.6.3 ROI-based robustness and cross-domain convergence analyses

To evaluate the robustness of whole-brain findings and examine potential overlap between abstract desirability and food valuation, ROI analyses were conducted in three *a priori* defined valuation-related regions: the anterior cingulate cortex (ACC), medial orbitofrontal cortex (mOFC), and medial prefrontal cortex (mPFC). ROIs were defined using anatomical atlas masks and restricted to voxels with valid functional coverage across participants. Due to incomplete functional coverage of the anatomical mOFC mask, a coverage-restricted mOFC mask was used.

Participant-level contrast estimates were extracted from each ROI for abstract desirability effects (OT, ET, and PT), combined abstract desirability (*Desire_Combined*), categorical food-value effects, and continuous food-value measures. ROI effects were summarized using mean parameter estimates, 95% confidence intervals, statistical tests where appropriate, and standardized effect sizes (Cohen’s *d*z). Multiple comparisons were controlled using Holm correction.

For the OT, ET, and PT contrasts, a formal conjunction-null analysis was conducted using a within-subject framework to test whether activation was consistently present across all three desirability domains.

To assess continuous neural representations of food value, trial-wise parametric modulation analyses were performed separately for the Hunger and Satiety Sessions. Individual food desirability ratings were entered as parametric modulators of stimulus-related BOLD responses. Because lower numerical ratings represented greater desirability, the sign of the parametric estimates was interpreted accordingly. Four models were examined: (1) *Hunger_value_slope*, assessing continuous food-value coding during hunger; (2) *Satiety_value_slope*, assessing food-value coding during satiety; (3) *Hunger_value > Satiety_value* coding, identifying stronger value-related responses during hunger; and (4) *Satiety_value > Hunger_value* coding, testing the reverse effect.

To examine convergence between abstract desirability and food valuation, ROI-level responses associated with abstract desirability were compared with food-value-related measures within the predefined valuation-related regions. The primary analysis compared *Desire_Combined* with *Hunger_value_slope* responses in the ACC, mOFC, and mPFC. Exploratory analyses further examined convergence with hunger-related modulation effects (*Hunger_value > Satiety_value*) and categorical food-value responses.

Additional identification-control analyses were conducted to distinguish desirability-related responses from general stimulus-processing demands. Desirability and identification judgments were directly compared using predefined whole-brain and ROI-level contrasts. Results of these control analyses are reported in the Supplementary materials.

#### 2.6.4 Spatial overlap and permutation-based robustness analyses

To further evaluate the spatial and individual-level correspondence between abstract desirability and food-value representations, complementary overlap and permutation-based analyses were conducted.

Spatial overlap between thresholded statistical maps was quantified using Dice and Jaccard coefficients. For two binary activation maps (A and B), Dice overlap was calculated as 2|A∩B|/(|A| + |B|), and Jaccard similarity was calculated as |A∩B|/|A∪B|. Overlap analyses were performed between the combined abstract desirability contrast (*Desire_Combined*) and food-value-related maps, including continuous hunger-related value coding, hunger-related value modulation, and categorical food-value contrasts. Overlap was quantified using the same thresholding schemes applied to the corresponding whole-brain maps.

Permutation tests were conducted to assess whether observed cross-domain correspondence exceeded chance expectations. Two complementary permutation approaches were applied. First, ROI-level sign consistency was evaluated by comparing the observed number of participants showing positive effects in both abstract desirability and food-value measures with a permutation-derived null distribution. Second, participant-level correlations between abstract desirability and food-value effects were compared against permutation-based null distributions generated by randomly shuffling participant labels.

## 3 Results

Behavioral and neuroimaging analyses were conducted to examine neural responses associated with abstract desirability evaluation and food incentive valuation, as well as their potential cross-domain convergence. We first evaluated behavioral performance and physiological-state manipulation, followed by whole-brain analyses of desirability and food-related responses. Finally, complementary convergence analyses using ROI-based approaches, spatial overlap metrics, and permutation tests were performed to assess the robustness of cross-domain correspondence.

### 3.1 Behavioral validation

To verify that the behavioral data supported subsequent neuroimaging analyses, we examined trial adequacy, response distributions, and the effectiveness of the physiological-state manipulation. Participant characteristics are summarized in Table 1A.

**Table 1:** Participant characteristics and behavioral validation of the DESIRE and HUNGER experiments.

**(A) Participant characteristics**	
*N*	10
Sex (male/female)	5/5
Age (years)	21.2 ± 3.6
BMI (kg/m^2^)	22.1 ± 4.4

**(B) DESIRE response distribution (%)**						
Domain	Desirable (%)	Indifferent (%)	Undesirable (%)	χ^2^(2)	*p*-Value	Kendall’s *W*
OT	34.84	26.85	38.31	5.60	0.061	0.28
ET	32.93	22.32	44.76	8.05	0.018	0.40
PT	30.18	27.54	42.28	4.20	0.122	0.21

**(C) Physiological-state manipulation**	
Condition	Fullness rating (1 = hungry, 10 = full)
DESIRE baseline	6.3 ± 1.16
Hunger before fasting	8.80 ± 1.14
Hunger Session (after fasting)	2.5 ± 0.53
Satiety	9.45 ± 0.60

**(D) Food classification**		
Category	Number of Trials	Percentage (%)
Hunger Food	659	58.3
Neutral Food	445	39.3
Irregular Food	27	2.4

Desirability percentages represent participant-level mean response proportions across the three semantic domains. Food classifications were based on within-participant changes in food desirability ratings between Hunger and Satiety Sessions. Hunger Foods indicated increased desirability during Hunger, Neutral Foods indicated unchanged desirability, and Irregular Foods indicated opposite-direction transitions in desirability between Hunger and Satiety Sessions. Fullness ratings significantly decreased after the fasting procedure (Wilcoxon signed-rank test, *W* = 0, *p* = 0.002) and were significantly higher during Satiety than Hunger Sessions (*W* = 0, *p* = 0.002).

In the DESIRE experiment, all participants contributed valid trials across the three response categories (*desirable, indifferent*, and *undesirable*) for each stimulus domain (objects, events, and persons), supporting separate modeling of response-based conditions in the first-level GLMs (Table 1B). No participant lacked any response category within any domain, indicating sufficient behavioral variation for estimating desirability-related neural responses.

The distribution of desirability responses showed that all three response categories were represented across the object, event, and person domains (Table 1B). Friedman tests revealed a significant response-category effect for event stimuli, χ^2^(2) = 8.05, *p* = 0.018, Kendall’s *W* = 0.40, whereas the corresponding effects for object and person stimuli did not reach significance. These findings indicate that the DESIRE task successfully elicited a range of subjective desirability judgments rather than a uniform preference pattern.

Behavioral measures confirmed successful manipulation of physiological state in the HUNGER experiment (Table 1C). Subjective fullness was assessed using a 10-point visual analogue scale (VAS; 1 = extremely hungry, 10 = extremely full). Fullness ratings significantly decreased following the fasting procedure, from 8.80 ± 1.14 before fasting to 2.50 ± 0.53 before the Hunger scan (Wilcoxon signed-rank test, *W* = 0, *p* = 0.002). Conversely, fullness ratings were significantly higher during the Satiety Session (9.45 ± 0.60) than during the Hunger Session (2.50 ± 0.53; *W* = 0, *p* = 0.002). The DESIRE session, conducted under a non-deprived baseline condition, showed intermediate fullness ratings before scanning (6.30 ± 1.16), consistent with its intended baseline motivational state.

Consistent with the physiological-state manipulation, food desirability responses differed between Hunger and Satiety Sessions. Compared with the Satiety Session, participants showed a significantly higher proportion of desirable (“want”) responses during Hunger (*p* = 0.001), a significantly lower proportion of undesirable responses (*p* = 0.001), and a significant change in indifferent responses (*p* = 0.027), indicating that physiological state modulated subjective food desirability.

We next examined participant-specific food classifications used for subsequent categorical analyses. Across all valid food trials, 659 trials (58.3%) were classified as Hunger Foods, 445 trials (39.3%) as Neutral Foods, and 27 trials (2.4%) as Irregular Foods (Table 1D, Supplementary Figure S3, and Supplementary Table S7). Thus, most food items showed increased desirability under Hunger, whereas opposite transitions were relatively rare. Two participants did not provide sufficient desirable Satiety trials for the categorical food-value contrast and were therefore excluded from analyses requiring this specific contrast, while being retained in continuous trial-wise analyses.

Together, these behavioral results suggest that (1) the DESIRE task generated sufficient variation in desirability judgments for neuroimaging modeling, and (2) the Hunger/Satiety manipulation successfully altered subjective food desirability, supporting the interpretation of subsequent neuroimaging analyses. Participant-level behavioral distributions and physiological-state ratings are provided in Supplementary Tables S6 and S7.

### 3.2 Neural correlates of abstract desirability evaluation

To examine neural responses associated with abstract desirability evaluation, we analyzed brain responses associated with desirable versus undesirable judgments across three semantic categories: object, event, and person. An exploratory whole-brain conjunction analysis was conducted to identify candidate regions showing spatially overlapping activation patterns across the three category-specific desirability contrasts.

At the exploratory whole-brain threshold (*p* < 0.01, uncorrected; cluster extent ≥ 20 contiguous voxels), the conjunction analysis revealed greater activation for desirable than undesirable judgments in medial frontal and cingulate regions, including the medial prefrontal cortex (mPFC), medial orbitofrontal cortex (mOFC), anterior cingulate cortex (ACC), and additional cingulate areas (Figure 2 and Table 2).

**Figure 2:**
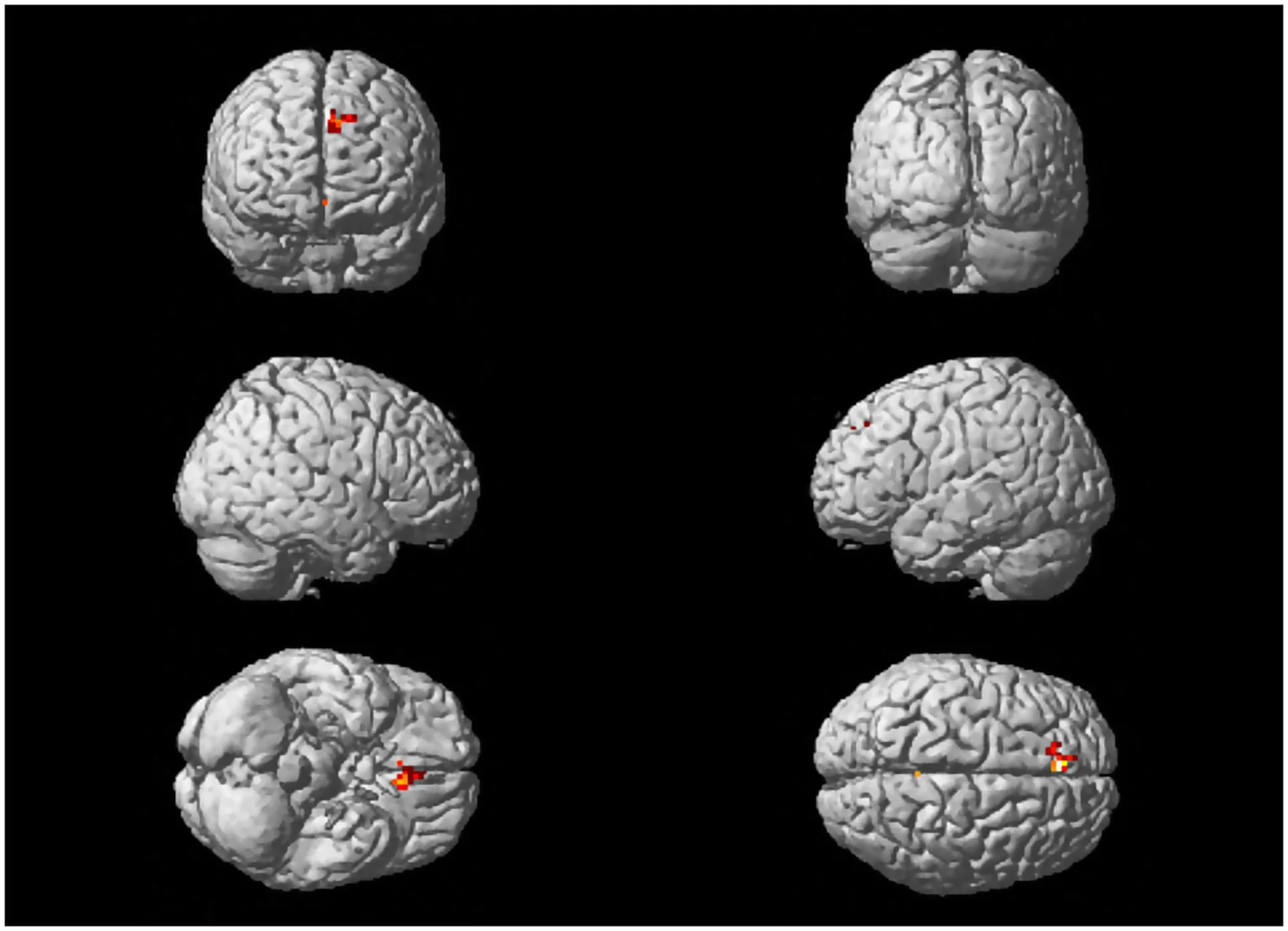
Neural correlates of abstract desirability evaluation across semantic domains. Statistical parametric maps showing brain regions exhibiting greater activation for *Desirable > Undesirable* judgments across the three semantic categories in the DESIRE experiment (objects, events, and persons). An exploratory whole-brain conjunction analysis was used to identify regions showing convergent responses across the three semantic categories. Results are displayed at a voxel-wise threshold of *p* < 0.01 (uncorrected) with a cluster-extent threshold of ≥20 contiguous voxels and are overlaid on a standard MNI template. Peak coordinates, cluster sizes, and statistical values are reported in Table 2. Brain images are displayed on a standard MNI template.

**Table 2:** Brain regions showing greater activation for desirable than undesirable judgments across object, event, and person categories in the DESIRE experiment.

**Brain region**	**BA**	**Cluster size (voxels)**	**Peak MNI**			***p* (unc.)**	***t*-value**
			***x***	***y***	***z***		
Frontal_Sup_Medial_L	9	37	-3	38	34	<0.0001	9.03
Frontal_Sup_L	一		−15	32	40	<0.0001	6.37
Frontal_Sup_Medial_L	一		−9	44	37	0.0006	4.68
Rectus_L	一	34	−6	26	−17	0.0006	4.69
Frontal_Med_Orb_R	11	13	6	26	−11	0.0006	4.60
Cingulum_Ant_L	32		−3	35	−8	0.002	3.82
Cingulum_Mid_R	一	20	6	−40	34	0.002	3.83
Cingulum_Mid_L	一		−3	−31	37	0.0056	3.18

Regions were identified by an exploratory whole-brain conjunction analysis across object, event, and person desirability contrasts. Because this analysis was exploratory, reported clusters should not be interpreted as confirmatory evidence. Peak coordinates are reported in Montreal Neurological Institute (MNI) space. BA, Brodmann area; cluster size indicates the number of contiguous suprathreshold voxels; L, left hemisphere; R, right hemisphere.

The identified regions have been implicated in subjective value representation, motivational evaluation, and preference-related processing. These findings indicate that exploratory conjunction analysis identified overlapping candidate activation patterns across semantic categories.

In addition, indifferent trials were modeled in the first-level GLM but were not included in the primary extreme-category contrasts. Additional ROI-level analyses involving indifferent trials are provided in Supplementary Table S8 and did not alter the interpretation of the primary findings.

### 3.3 Neural correlates of baseline food incentive value

To characterize neural responses associated with baseline food incentive value, we examined brain activity associated with desirable versus undesirable food stimuli across the Hunger and Satiety Sessions. This contrast was designed to capture individual differences in food motivational value independent of the transient changes associated with physiological state.

At the exploratory whole-brain threshold (*p* < 0.001, uncorrected; cluster extent ≥ 20 contiguous voxels), desirable food stimuli elicited greater activation than undesirable food stimuli in medial frontal and orbitofrontal regions, including the medial orbitofrontal cortex, medial frontal cortex, and anterior cingulate regions (Figure 3 and Table 3).

**Figure 3:**
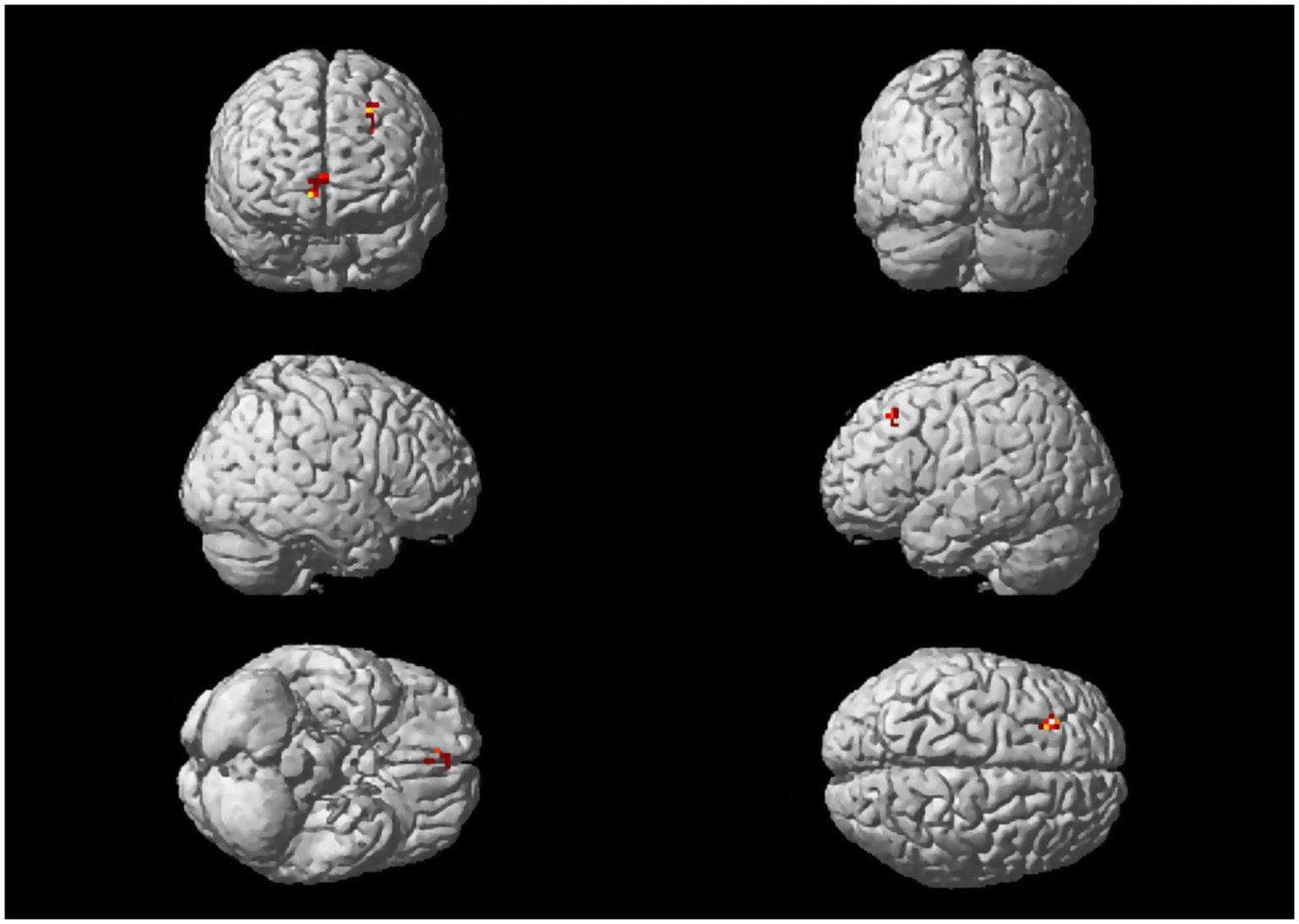
Neural correlates of baseline food incentive value across Hunger and Satiety States. Statistical parametric maps showing brain regions exhibiting greater activation for *Desirable > Undesirable* food stimuli across the Hunger and Satiety Sessions in the HUNGER experiment. Contrast images from both physiological states were combined at the second level to estimate baseline food incentive-value responses. Results are displayed at a voxel-wise threshold of *p* < 0.001 (uncorrected) with a cluster-extent threshold of ≥20 contiguous voxels and are overlaid on a standard MNI template. Peak coordinates and statistical values are reported in Table 3.

**Table 3:** Brain regions showing greater activation for *Desirable > Undesirable* food stimuli across Hunger and Satiety Sessions.

**Brain regions**	**BA**	**Cluster size (voxels)**	**Peak MNI**			***p* (unc.)**	***t*-value**
			***x***	***y***	***z***		
Frontal_Med_Orb_R (aal)	10	38	9	50	−2	<0.0001	7.67
Frontal_Med_Orb_R	10		6	44	−8	0.0001	5.84
Frontal_Sup_Medial_R	10		3	53	7	0.0002	5.43
Frontal_Mid_L	8	20	−24	26	46	<0.0001	7.07
Frontal_Mid_L	9		−27	32	37	0.0004	4.96
Cingulum_Ant_R	32	33	6	35	16	<0.0001	6.35
Cingulum_Ant_L	一		0	23	28	0.0001	5.77
Cingulum_Ant_L	一		−3	23	19	0.0003	5.26

Regions associated with food incentive value were identified using a whole-brain random-effects analysis across both physiological states. Peak coordinates are reported in Montreal Neurological Institute (MNI) space. BA, Brodmann area; cluster size indicates the number of contiguous suprathreshold voxels.

The identified regions have been implicated in subjective value representation, reward-related evaluation, and motivational significance. These findings suggest that baseline food incentive value engages valuation-related neural regions involved in representing the motivational relevance of biologically important stimuli.

Because this contrast averaged across Hunger and Satiety states, it reflects general food incentive value processing across physiological states rather than hunger-induced modulation. State-dependent changes in food desirability were examined separately in the subsequent analysis.

### 3.4 Neural responses associated with hunger-related increases in food desirability

To examine neural responses associated with hunger-related changes in food desirability, we compared participant-specific Hunger Foods with participant-specific Neutral Foods within the Hunger Session. This analysis was independent of the baseline food incentive-value contrast reported in the previous section because it was based on cross-session rating changes rather than overall desirability level. Hunger Foods were defined as participant-by-item observations for which the Hunger Session rating was lower than the Satiety Session rating, indicating increased willingness to consume these items under hunger. Neutral Foods were defined as items showing identical desirability ratings across the two physiological states, indicating stable food desirability.

At the exploratory whole-brain threshold (*p* < 0.005, uncorrected; cluster extent ≥ 10 contiguous voxels), Hunger Foods elicited greater activation than Neutral Foods in the left inferior orbitofrontal cortex (OFC), together with additional clusters involving supplementary motor, precentral, fusiform, cerebellar, and occipital regions within the Hunger Session (Figure 4 and Table 4).

**Figure 4:**
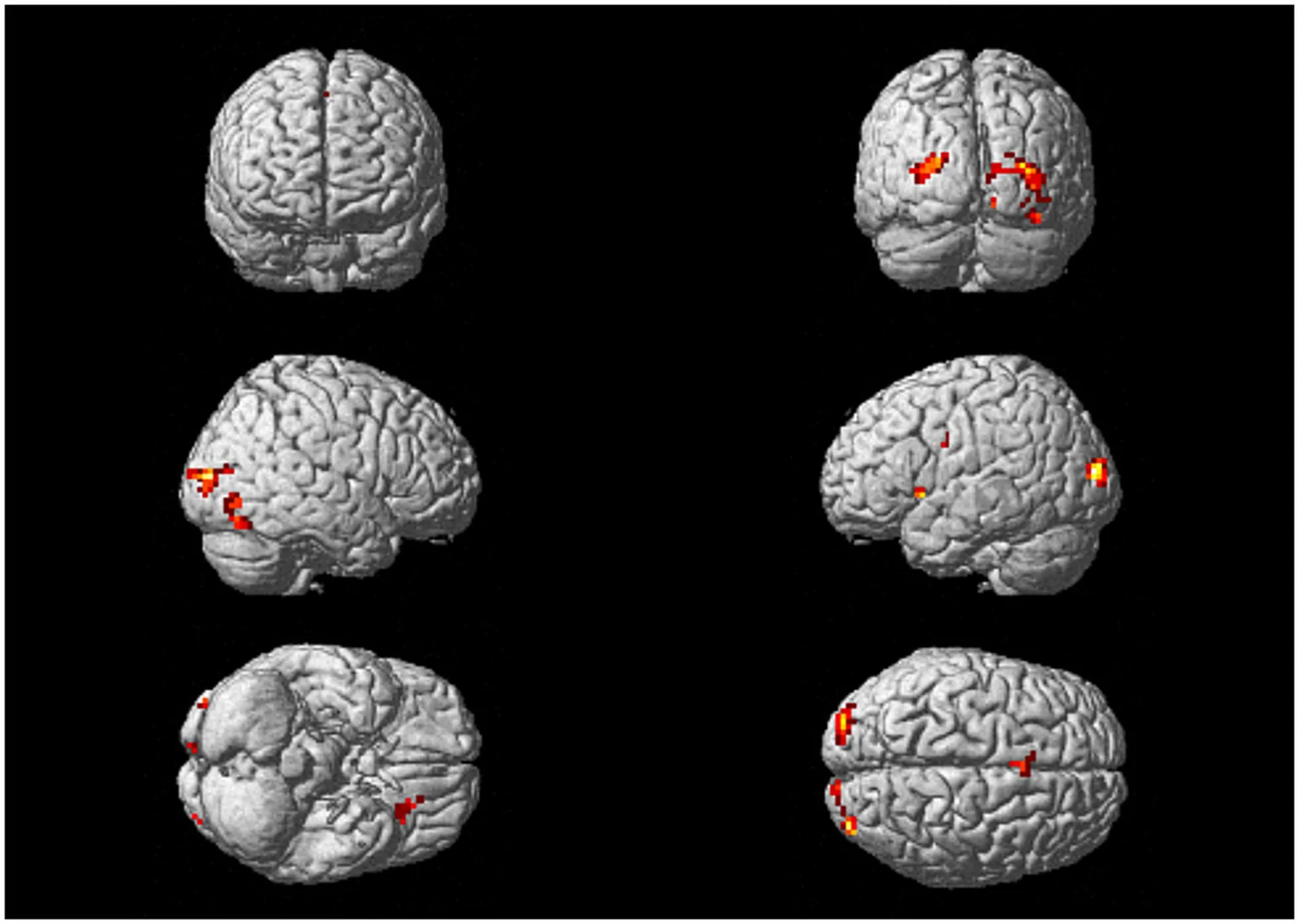
Neural responses associated with hunger-related increases in food desirability. Statistical parametric maps showing brain regions exhibiting greater activation for Hunger Foods than Neutral Foods within the Hunger Session of the HUNGER experiment. Results are displayed at a voxel-wise threshold of *p* < 0.005 (uncorrected) with a cluster extent threshold of ≥10 contiguous voxels, and are overlaid on a standard MNI template. Peak coordinates and statistical values are reported in Table 4.

**Table 4:** Brain regions showing greater activation for participant-specific Hunger Foods than Neutral Foods during the Hunger Session.

**Brain regions**	**BA**	**Cluster size (voxels)**	**Peak MNI**			***p* (unc.)**	***t*-value**
			**x**	**y**	**z**		
Unlabeled cluster	一	26	−30	14	1	0.0001	5.71
Supp_Motor_Area_L	一	39	−9	8	46	0.0003	5.26
Supp_Motor_Area_L	一		−3	8	58	0.0012	4.18
Supp_Motor_Area_R	6		3	14	49	0.0031	3.56
Cerebelum_Crus1_R	一	97	39	−73	−20	0.0003	5.25
Fusiform_R	一		33	−46	−17	0.0005	4.83
Cerebelum_6_R	一		27	−64	−17	0.0005	4.73
Frontal_Inf_Orb_L	一	18	−24	29	−11	0.0003	5.16
Unlabeled cluster	一		−21	38	−8	0.0034	3.49
Occipital_Mid_L	一	54	−27	−94	13	0.0004	4.93
Occipital_Mid_R	18	75	36	−88	10	0.0004	4.87
Occipital_Mid_R	19		36	−94	1	0.0011	4.22
Cuneus_R	一		18	−97	10	0.0012	4.18
Unlabeled cluster	一	12	−33	−4	−11	0.0005	4.75
Precentral_L	6	12	−48	−4	31	0.0008	4.46
Precentral_L	一		−42	−4	25	0.0009	4.34
Lingual_R	一	19	12	−82	−11	0.0009	4.33
Lingual_R	18		18	−76	−8	0.0024	3.71

Regions were identified using a whole-brain random-effects analysis comparing participant-specific Hunger Foods and Neutral Foods within the Hunger Session. Hunger Foods were defined based on individual increases in desirability from Satiety to Hunger. Peak coordinates are reported in Montreal Neurological Institute (MNI) space. BA, Brodmann area; cluster size indicates the number of contiguous suprathreshold voxels.

This activation pattern suggests that food items showing increased desirability under hunger were associated with enhanced responses in valuation-related, visual-perceptual, and sensorimotor/action-related regions. These additional regions may reflect increased attentional and action-related processing toward food stimuli that become more motivationally relevant under physiological need.

Because this contrast was based on participant-specific behavioral classification, the observed activation pattern is interpreted as neural responses associated with hunger-related changes in food desirability rather than as a pure measure of hunger-specific valuation enhancement. In addition, because Hunger Foods may differ from Neutral Foods in current desirability level during the Hunger Session, this contrast should be interpreted cautiously.

### 3.5 Cross-domain convergence analyses

The exploratory whole-brain analyses identified partially similar candidate valuation-related regions in abstract desirability and food-value contrasts. However, spatially similar activation patterns do not necessarily indicate a statistically reliable shared neural representation. We therefore conducted a series of complementary convergence analyses.

#### 3.5.1 Convergence across abstract desirability domains

The exploratory whole-brain conjunction analysis described above identified candidate regions showing overlapping responses across object, event, and person desirability judgments. However, spatial overlap in exploratory activation maps does not necessarily indicate a statistically reliable common effect across domains. Therefore, to determine whether these overlapping patterns reflected statistically reliable common responses across domains, we conducted a formal within-subject conjunction-null analysis. No clusters survived whole-brain FWE correction, and no suprathreshold clusters were observed even at an exploratory threshold of *p* < 0.001 (uncorrected), indicating that the exploratory overlap did not represent a robust domain-general neural response pattern in the present sample.

We further examined convergence within independently defined valuation-related ROIs (ACC, mOFC, and mPFC) using participant-level positive conjunction analyses across the three desirability domains. No ROI showed significant positive conjunction effects (ACC: *p* = 0.883; mOFC: *p* = 0.984; mPFC: *p* = 0.992; Supplementary Table S4). Descriptively, effect estimates showed heterogeneous patterns across domains, with small positive tendencies for object and event desirability in medial orbitofrontal regions but relatively weaker or negative responses for person desirability. Together, these findings suggest that although exploratory whole-brain mapping revealed candidate overlapping regions, stricter conjunction-based and ROI-level analyses did not provide evidence for a statistically robust common neural substrate shared across object, event, and person desirability judgments.

#### 3.5.2 Convergence between abstract desirability and food valuation

To examine whether abstract desirability and food valuation shared common neural representations beyond categorical desirability contrasts, and to avoid potential dependence on arbitrary categorical classifications, we first assessed continuous food-value coding using trial-wise parametric modulation analyses. Food desirability ratings were entered as continuous modulators of stimulus-related BOLD responses separately for the Hunger and Satiety Sessions.

Four whole-brain food-value models were examined: (1) *Hunger_value_slope*, reflecting continuous parametric modulation of BOLD responses by subjective food desirability during Hunger Session; (2) *Satiety_value_slope*, reflecting continuous food-value coding during the Satiety Session; (3) *Hunger_value_slope* > *Satiety_value_slope*, testing whether value-related responses were enhanced during Hunger; and (4) *Satiety_value_slope* > *Hunger_value_slope*, testing the reverse effect. None of these four models revealed significant effects after whole-brain FWE correction. In the predefined ACC, mOFC, and mPFC ROIs, no trial-wise food-value effects survived Holm correction for multiple comparisons (Supplementary Table S3). Descriptively, *Hunger_value_slope* estimates were negative across all three ROIs. Although nominal uncorrected effects were observed in the ACC (*p* = 0.034) and mOFC (*p* = 0.054), neither effect survived Holm correction.

We next directly examined whether abstract desirability and food valuation converged within the same valuation-related regions. Abstract desirability was represented by the combined desirability contrast: *Desire_Combined* = (*OT+ET+PT*)/3. The primary convergence analysis compared *Desire_Combined* with *Hunger_value_slope* responses in the ACC, mOFC, and mPFC, while secondary analyses examined convergence with *Hunger_value_slope* > *Satiety_value_slope* and categorical food-value responses (*Desirable > Undesirable*).

Across these ROI-level comparisons, none of the correlation-based or sign-consistency tests provided significant evidence for cross-domain convergence (Table 5). Figure 5 illustrates the ROI-level comparisons between abstract desirability and food-value measures.

**Table 5:** ROI-based convergence between abstract desirability and food valuation.

(A) Desire_Combined vs Hunger_value_slope					
**ROI**	**Pearson *r***	***p***	**Permutation *p* (*r*)**	**Both-positive *N***	**Permutation *p* (sign)**
ACC	0.199	0.581	0.577	2	0.34
mOFC	0.605	0.064	0.062	2	0.07
mPFC	0.574	0.083	0.083	1	0.70

(B) Desire_Combined vs Hunger_Minus_Satiety_Slope					
**ROI**	**Pearson *r***	***p***	**Permutation *p* (*r*)**	**Both-positive *N***	**Permutation *p* (sign)**
ACC	−0.192	0.595	0.605	3	0.66
mOFC	0.319	0.369	0.377	3	0.17
mPFC	−0.119	0.744	0.746	3	0.30

(C) Desire_Combined vs Categorical_Food					
**ROI**	**Pearson *r***	***p***	**Permutation *p* (*r*)**	**Both-positive *N***	**Permutation *p* (sign)**
ACC	−0.682	0.062	0.053	1	0.83
mOFC	0.065	0.879	0.887	0	1.00
mPFC	0.081	0.848	0.846	1	0.64

Desire_Combined represents the averaged desirability contrast across object, event, and person domains. Hunger_value_slope represents continuous food-value coding during the Hunger Session. Hunger_Minus_Satiety_Slope represents the difference between food-value coding during Hunger and Satiety. Categorical_Food represents the Desirable > Undesirable food contrast. Correlation-based and sign-consistency permutation tests did not provide evidence for significant cross-domain convergence after permutation-based significance testing. **(A,B)** Included all 10 participants; **(C)** included 8 participants because two participants lacked sufficient trials for the categorical food-value contrast.

**Figure 5:**
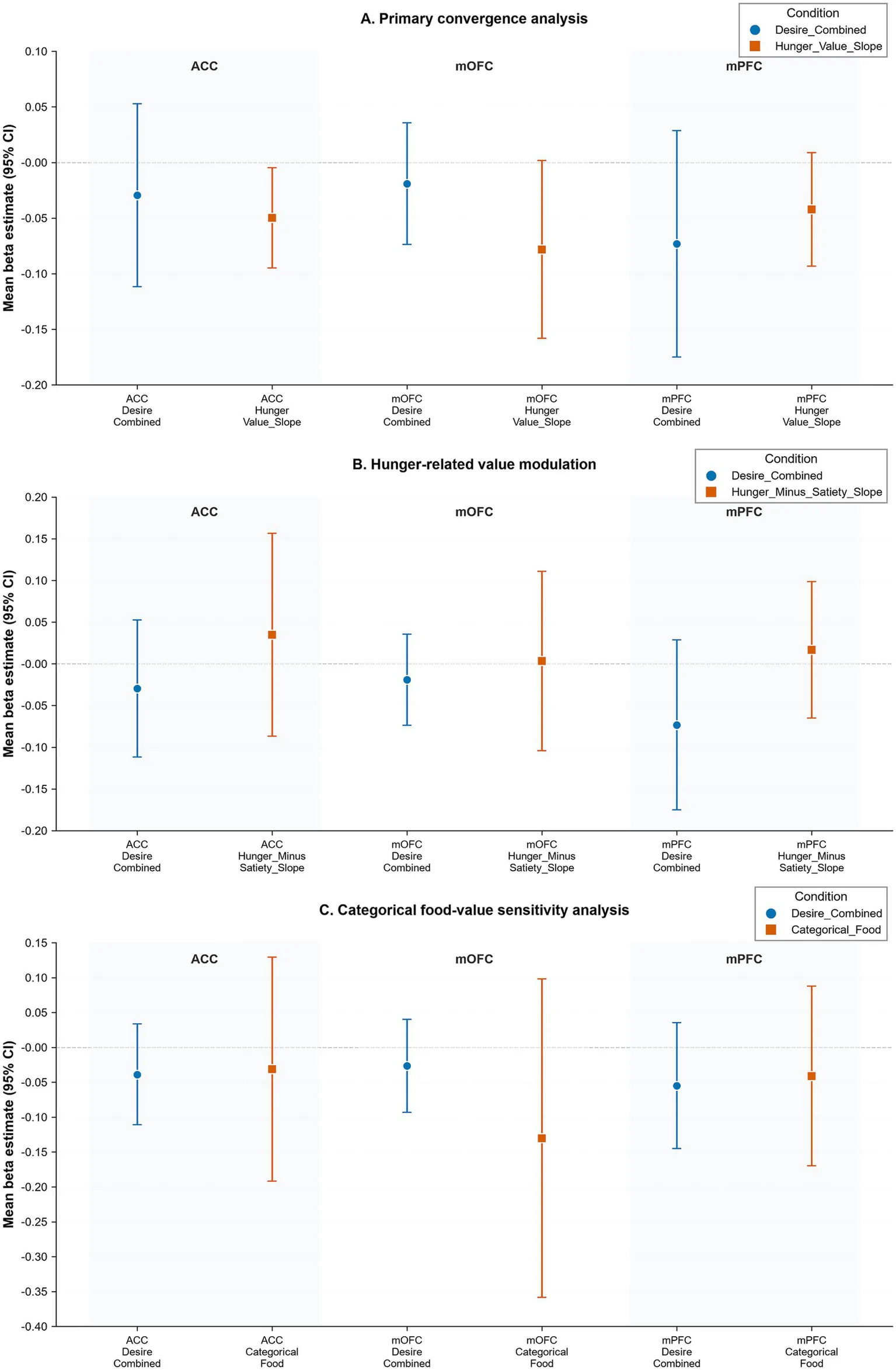
ROI-level comparison of abstract desirability and food valuation effects. Mean parameter estimates and 95% confidence intervals are shown for the ACC, mOFC, and mPFC. **(A)** Shows the primary comparison between *Desire_Combined* and continuous *Hunger_value_slope* coding. **(B)** Shows secondary comparisons between *Desire_Combined* and Hunger-related value modulation (*Hunger_value_slope* > *Satiety_value_slope*). **(C)** Shows a sensitivity analysis comparing *Desire_Combined* with *Categorical_Food* value responses. Error bars indicate 95% confidence intervals. None of the ROI-level effects survived Holm correction for multiple comparisons.

In the primary analysis, *Desire_Combined* and *Hunger_value_slope* both showed non-positive mean estimates across ACC, mOFC, and mPFC. In the secondary analyses, *Hunger-minus-Satiety* food-value slopes were close to zero or weakly positive, whereas categorical food-value effects were small and non-significant across all three ROIs. Thus, although abstract desirability and food valuation showed partially similar candidate valuation-related activation patterns at the exploratory whole-brain level, ROI-based analyses did not provide evidence for a statistically reliable shared positive response pattern across domains.

#### 3.5.3 Whole-brain spatial overlap and permutation-based robustness analyses

To further assess whether abstract desirability and food valuation showed spatial correspondence beyond ROI-level effects, whole-brain overlap between thresholded statistical maps was quantified using Dice and Jaccard coefficients. Three comparisons were examined: *Desire_Combined* versus *Hunger_value_slope*, *Hunger_value_slope* > *Satiety_value_slope*, and *Categorical_Food* value responses.

Across all comparisons and exploratory thresholding schemes, no suprathreshold voxel overlap was observed between abstract desirability and food-value maps (Supplementary Table S9), indicating that exploratory activation patterns from the two domains did not show measurable spatial correspondence at the thresholded map level.

Permutation-based analyses further tested whether observed participant-level correspondence exceeded chance expectations. Neither ROI-level correlations nor the number of participants showing positive effects across abstract desirability and food-value measures exceeded permutation-derived null distributions in the ACC, mOFC, or mPFC (Table 5 and Supplementary Table S10). Together, these results did not provide evidence for cross-domain convergence beyond chance expectations.

## 4 Discussion

The present study compared cross-category desirability evaluation under a non-deprived baseline condition with food incentive evaluation under experimentally manipulated Hunger and Satiety states. Exploratory whole-brain analyses identified candidate similarity in medial valuation-related regions across the two motivational domains, but formal conjunction, ROI-based convergence, spatial overlap, and permutation analyses did not support a statistically robust shared neural representation. These findings suggest that valuation-related processing may show partial generalization across motivational contexts while remaining sensitive to semantic content and physiological-state regulation.

### 4.1 Abstract desirability recruits valuation-related processes but does not reflect a unitary domain-general representation

One central aim of the present study was to determine whether abstract desirability evaluation relies on neural systems commonly implicated in motivational valuation. Across three semantically distinct categories—objects, events, and persons—desirability judgments were associated with activation patterns involving medial frontal and cingulate regions, including the medial prefrontal cortex (mPFC), medial orbitofrontal cortex (mOFC), and anterior cingulate cortex (ACC). These regions have consistently been implicated in representing subjective value, motivational significance, and goal-related evaluation across diverse domains (Kennerley and Walton, 2011; Bartra et al., 2013; Knudsen and Wallis, 2022; Rolls, 2023). The present findings therefore suggest that evaluating desirability across semantically heterogeneous targets involves neural systems previously implicated in motivational value processing, rather than relying only on category-specific semantic or perceptual representations.

The involvement of medial frontal valuation-related regions during abstract desirability judgment is consistent with previous work showing that the mPFC and OFC contribute to the computation of subjective value and preference across different types of decisions and rewards (Bartra et al., 2013; Redish et al., 2016; Rolls, 2023; Qiu et al., 2024). Similarly, the ACC has been associated with motivational relevance, action selection, and the evaluation of goal-directed behavior (Kennerley and Walton, 2011; Foinikianaki et al., 2025). In the present study, these regions were observed when participants evaluated how much they wanted to obtain an object, experience an event, or become a person represented in an image. This pattern is consistent with the possibility that motivational evaluation may operate not only for externally delivered rewards or biologically relevant incentives, but also for desired targets whose motivational relevance is not directly tied to an experimentally induced homeostatic need.

However, the present findings also refine the interpretation of this valuation-related involvement. Although the exploratory whole-brain conjunction analysis revealed candidate overlapping activation patterns across object, event, and person desirability judgments, the formal conjunction-null analysis did not identify statistically reliable common activation across all three domains. Moreover, ROI-based consistency analyses within the ACC, mOFC, and mPFC did not provide evidence for a significant positive conjunction across object, event, and person desirability responses. These findings suggest that while valuation-related regions may contribute to abstract desirability evaluation, the underlying neural processes are unlikely to reflect a single, fully domain-general representation shared equally across different semantic categories.

One possible interpretation is that abstract desirability evaluation relies on partially shared valuation-related computations whose expression depends on the specific nature of the desired target and the cognitive processes required to evaluate it. Objects, events, and persons differ substantially in their semantic structure, temporal characteristics, and behavioral implications. For example, wanting to obtain an object may involve possession-related value, wanting to participate in an event may involve anticipated experience, and wanting to become a person may involve self-relevant or identity-related evaluation. Although these different forms of desire may recruit overlapping motivational systems, they may also engage partially distinct neural processes related to semantic context, personal relevance, and future-oriented simulation.

This interpretation is consistent with broader accounts of valuation systems suggesting that medial frontal and orbitofrontal regions support flexible evaluation across multiple domains rather than encoding a single fixed value representation. The present findings therefore support a more nuanced view: abstract motivational cognition appears to draw upon valuation-related neural mechanisms, but these mechanisms are distributed and context-sensitive rather than organized as a unitary domain-general neural code.

Importantly, the identification-based runs were not included as primary task contrasts in the main analyses but were subsequently examined in supplementary identification-control analyses. The observed activation patterns should be interpreted as neural correlates associated with desirability evaluation rather than as responses uniquely isolated from all non-motivational processes, such as semantic processing, stimulus familiarity, or decision-related demands. Future studies combining desirability and identification judgments within the same analytical framework, together with larger samples and more stringent statistical approaches, will be necessary to further clarify which aspects of these medial frontal responses specifically reflect motivational valuation.

### 4.2 Food incentive value and hunger-related changes reveal state-dependent motivational processing

The HUNGER experiment provided a physiological reference for interpreting motivational valuation processes by examining food desirability under different internal states. Consistent with previous studies showing that medial frontal and orbitofrontal regions contribute to food valuation and reward-related evaluation (Berridge, 2004; Siep et al., 2009; Shimizu et al., 2010; Bartra et al., 2013; Huerta et al., 2014), the baseline food incentive-value contrast (*desirable > undesirable* food stimuli across Hunger and Satiety Sessions) identified activation patterns involving medial orbitofrontal, medial frontal, and anterior cingulate regions. These findings indicate that food-related motivational value engages neural systems that overlap with those involved in broader subjective value computation.

The baseline food-value analysis served an important role in the present study because it provided a physiological-domain comparison for abstract desirability evaluation. The observation that both abstract desirability judgments and baseline food incentive-value evaluation involved medial valuation-related regions suggests that different motivational domains may partially rely on common evaluative processes. However, this overlap should not be interpreted as evidence that abstract desire and food motivation are represented by identical neural mechanisms. Rather, these findings suggest that medial frontal valuation-related systems may support a more general process of evaluating motivational relevance across different types of desired targets.

Beyond baseline food value, the present study further examined how physiological state alters the motivational significance of specific food stimuli. Instead of assuming that all food stimuli become equally more desirable during hunger, we used participant-specific changes in desirability ratings between Hunger and Satiety Sessions to identify Hunger Foods—food items whose desirability increased under hunger—and compared them with Neutral Foods whose desirability remained stable across states. This behaviorally grounded approach allowed the neural analysis to focus on food stimuli that became specifically more motivationally relevant under physiological need.

The *Hunger Foods > Neutral Foods* contrast revealed a broader pattern involving the left inferior orbitofrontal cortex, visual-perceptual regions, cerebellar regions, and sensorimotor areas. The involvement of inferior OFC is consistent with the role of orbitofrontal regions in updating the incentive value of food stimuli according to current physiological states and motivational needs (Siep et al., 2009). However, the additional engagement of fusiform, occipital, lingual, supplementary motor, precentral, and cerebellar regions suggests that hunger-related increases in food desirability may involve more than a simple amplification of value signals. Instead, physiological need may enhance the perceptual salience, attentional priority, and action relevance of food stimuli that become particularly desirable under hunger (Montagrin et al., 2021; Smith and Grueter, 2022).

This interpretation is consistent with models of motivated behavior suggesting that wanting involves not only the representation of reward value but also processes supporting approach preparation and goal-directed action (Berridge, 2004; Yager and Robinson, 2010; Sokol-Hessner et al., 2015; Lopez-Gamundi et al., 2021; Messimeris et al., 2023). When physiological states increase the relevance of a stimulus, neural systems involved in perception and action may interact with valuation-related regions to facilitate responses toward motivationally significant targets. Thus, the present findings suggest that hunger-related changes in food desirability reflect a state-dependent reorganization of motivational processing, in which valuation-related updating is accompanied by enhanced perceptual and action-related responses.

Importantly, the baseline food-value and *Hunger Foods > Neutral Foods* contrasts capture different aspects of food motivation. The former reflects general differences in food incentive value across physiological states, whereas the latter reflects individual-specific changes in desirability associated with hunger. The partially distinct neural patterns observed in these analyses suggest that food motivation is not represented by a single static valuation signal; rather, it emerges from interactions between subjective value, physiological state, perceptual relevance, and behavioral readiness.

### 4.3 Cross-domain convergence and implications for embodied motivational cognition

A central theoretical motivation of the present study was to examine whether abstract motivational concepts may rely, at least partly, on neural systems that also support biologically grounded motivational states. The comparison between abstract desirability evaluation and hunger-related food valuation was motivated by the proposal that some abstract concepts are grounded in systems originally evolved for bodily regulation and action control. Within this framework, the primary metaphor DESIRE IS HUNGER is theoretically distinctive because its source domain is not derived primarily from external sensory experience, but from an internal physiological state. In contrast to many extensively studied primary metaphors whose source domains are grounded in exteroceptive experiences such as vision, touch, or spatial relations, hunger represents an interoceptive motivational state that directly reflects bodily needs (Gallese and Lakoff, 2005; García-García et al., 2013; Citron and Goldberg, 2014; Desai, 2022; Barca et al., 2026). Importantly, this comparison should not be understood as implying a categorical separation between an entirely “abstract” DESIRE system and an entirely “physiological” food system. Food evaluation itself contains general valuation processes, and generalized desirability may also be influenced by bodily state. The experimentally relevant distinction is that physiological state was explicitly manipulated and homeostatically relevant in the HUNGER experiment, but not in the DESIRE experiment.

The present findings provide a nuanced perspective on this possibility. Exploratory whole-brain analyses identified partially overlapping medial frontal and cingulate valuation-related regions during abstract desirability evaluation and baseline food incentive-value evaluation. This spatial similarity is consistent with the possibility that abstract desires and physiological incentives may recruit partially shared valuation-related processes. Such overlap is theoretically compatible with grounded cognition accounts proposing that conceptual knowledge is not entirely independent from the systems involved in perception, action, and bodily states (Friedrich et al., 2025; Barsalou, 2026). It is also consistent with neural reuse perspectives, which suggest that evolutionarily older neural systems supporting basic biological functions may be recruited and adapted for more complex cognitive operations (Anderson, 2010, 2014; Kiverstein, 2020).

However, the present results also place important constraints on strong interpretations of this hypothesis. Although exploratory activation patterns suggested candidate convergence, formal conjunction-null analyses, ROI-based consistency analyses, whole-brain overlap analyses, and permutation-based tests did not provide evidence for a statistically robust shared neural representation across abstract desirability and food valuation. Therefore, the current findings do not indicate that abstract desire and hunger are encoded by an identical neural mechanism or that abstract motivational concepts can be reduced to physiological drives. Instead, the results suggest that these two motivational contexts may partially rely on common evaluative processes while retaining domain-specific and context-dependent neural characteristics.

This distinction is important for theories of embodied cognition and conceptual metaphor. A common assumption in embodied approaches is that abstract concepts may partially inherit structure from sensorimotor and bodily systems through repeated experience and neural reuse (Barsalou, 2008; Anderson, 2010; Kiverstein, 2020). However, embodiment does not necessarily imply one-to-one neural correspondence between a bodily source domain and an abstract target domain. The present findings support a weaker but theoretically meaningful interpretation: motivational concepts may be partially grounded in shared valuation mechanisms that also contribute to physiological motivation, while the specific neural expression of these concepts depends on semantic context, personal relevance, and current bodily state.

The present results therefore provide a more differentiated view of the relationship between abstract desire and hunger. Hunger-related food motivation showed broader state-sensitive responses involving not only valuation-related regions but also perceptual and action-related systems, suggesting that physiological needs can modify how motivationally relevant stimuli are perceived and acted upon. In contrast, abstract desirability judgments were assessed without explicit manipulation of an acute physiological need state. This pattern suggests that abstract desires may preserve elements of motivational evaluation that are shared with biological drives, while being transformed by higher-level cognitive processes such as semantic representation, future-oriented simulation, and goal evaluation.

From the perspective of Conceptual Metaphor Theory, the present findings are therefore compatible with the possibility that DESIRE IS HUNGER reflects partial grounding of abstract motivational concepts in interoceptive valuation systems rather than a direct neural mapping between hunger and desire. Previous accounts of conceptual metaphors have emphasized that abstract concepts may emerge through systematic mappings from embodied experience (Grady, 1997; Lakoff and Johnson, 1999; Pecher et al., 2011). The current results extend this discussion by suggesting that, for motivational concepts, the relevant bodily basis may involve valuation-related processes associated with internal physiological states and their associated valuation systems. Nevertheless, because the present analyses did not demonstrate statistically robust neural convergence, the findings should be interpreted as preliminary evidence for shared motivational architecture rather than direct neural confirmation of a conceptual metaphor mapping.

Together, the present findings suggest that abstract motivational cognition may be supported by a partially shared but heterogeneous valuation architecture. Rather than revealing a single neural code linking desire and hunger, the observed pattern indicates that motivational evaluation may emerge from interactions between conserved valuation systems and domain-specific cognitive processes. This perspective provides a framework for understanding how bodily states may contribute to abstract motivational concepts while preserving the flexibility and complexity of human desires.

### 4.4 Methodological considerations and future directions

Several methodological considerations should be taken into account when interpreting the present findings.

First, the relatively small sample size (*N* = 10) limits statistical stability and generalizability. Although the within-subject design reduced variability associated with individual differences and allowed direct comparison between abstract desirability and food valuation within the same participants, the present findings should be considered exploratory. Future studies with larger samples will be necessary to determine the reliability and population-level generalizability of the observed patterns.

Second, the whole-brain analyses were conducted using exploratory thresholds, and several reported activation patterns should therefore be interpreted cautiously. The present study addressed this limitation by incorporating complementary robustness analyses, including ROI-based analyses, formal conjunction tests, spatial overlap analyses, and permutation-based assessments. Nevertheless, future work using preregistered hypotheses and larger samples will be important for testing the reproducibility of the identified candidate regions.

Third, although the present task was designed to emphasize motivational desirability (“wanting”) rather than hedonic liking, liking responses were not independently measured. Therefore, the current findings cannot completely exclude contributions from hedonic evaluation or related affective processes. Future studies directly measuring both wanting and liking would help clarify the extent to which valuation-related activity reflects motivational desire specifically.

Fourth, the present study did not collect objective metabolic measures of physiological state. Although fasting procedures, subjective fullness ratings, and behavioral changes in food desirability supported the effectiveness of the Hunger/Satiety manipulation, future studies incorporating physiological markers such as metabolic or hormonal measures may provide a more comprehensive characterization of the relationship between bodily states and neural valuation processes.

Finally, the present analyses focused primarily on spatial convergence between motivational domains rather than directly testing whether the same neural representations support abstract desire and food motivation. Future studies applying multivariate pattern analysis, representational similarity analysis, connectivity-based approaches, or computational modeling may help determine whether observed overlap reflects shared computations, partially reused neural resources, or functionally distinct processes within neighboring valuation-related regions.

Together, these future directions will help clarify how biological motivational states and abstract motivational concepts interact within human valuation systems.

## Data Availability

The raw data supporting the conclusions of this article will be made available by the authors, without undue reservation.
