# Supplementary material for: Exploring partially shared valuation-related neural responses between abstract desirability and food incentive evaluation
Source: Front Hum Neurosci. 2026 Sep 30;20:1909419. doi: 10.3389/fnhum.2026.1909419 (PMC13661570; doi:10.3389/fnhum.2026.1909419)
Supplement: Supplementary file 1 [file Data_Sheet_1.PDF]

Supplementary Material

Exploring Partially Shared Valuation-Related Neural Responses Between Abstract Desirability and Food Incentive Evaluation

Supplementary Methods S1. Stimulus Validation and Selection Procedure

S1.1 DESIRE stimulus validation and selection

To establish a stimulus set capable of eliciting motivational desirability judgments across diverse semantic domains, an initial pool of 1,245 images was collected from three categories: objects, events, and persons. The stimulus selection procedure was designed to maximize variability in subjective desirability while minimizing ambiguity, extreme emotional content, and category-specific biases.

Because the present study focused on motivational desirability (“wanting”) rather than hedonic pleasure (“liking”), participants in the validation study received detailed instructions distinguishing these two components of reward processing (Berridge, 2009; Grabenhorst and Rolls, 2011; Bosulu et al., 2022). Participants were informed that wanting refers to the motivation or desire to obtain, experience, or pursue something, whereas liking refers to the pleasure associated with experiencing or consuming it (Berridge and Robinson, 2016; Morales and Berridge, 2020; Nguyen et al., 2021). To facilitate understanding, participants were provided with examples illustrating situations in which wanting and liking may diverge. This distinction was emphasized throughout the validation procedure.

An independent behavioral validation study was conducted with 41 participants who did not participate in the fMRI experiment. Before rating the stimuli, participants reported their current fullness state using a 10-point visual analogue scale (VAS; 1 = extremely hungry, 10 = extremely full). Participants then completed three validation runs corresponding to the three stimulus categories (objects, events, and persons). After practice trials, participants evaluated each image using a 10-point desirability scale (1 = least desirable, 10 = most desirable) implemented in E-Prime. The behavioral validation procedure and stimulus selection workflow are illustrated in Supplementary Figure S1.

【Insert Supplementary Figure S1 here.】

Stimulus selection was performed using a two-stage procedure. First, images were filtered according to category-specific mean desirability thresholds. Second, coefficient of variation (CV = standard deviation / mean desirability rating) was used to identify stimuli with higher rating consistency across participants. Images with similar, ambiguous, or potentially uncomfortable content were further excluded through manual inspection.

The category-specific selection criteria and the number of retained images at each selection stage are summarized in Supplementary Table S1. The final fMRI stimulus set included 24 images from each desirability level (desirable, indifferent, and undesirable) within each semantic category, resulting in 72 stimuli per category and 216 stimuli in total.

Supplementary Table S1. Normative rating thresholds and stimulus selection procedure for the DESIRE experiment

Stimulus Category	Desirability Level	Mean Rating Threshold (Round 1)	Images Retained (Round 1)	CV Criterion (Round 2)	Images Retained (Round 2)	Final Images (fMRI)
Object	Desirable	>7.5	65	<0.3	53	24
	Intermediate	4<OI<6	84	<0.6	65	24
	Undesirable	<2.5	116	<0.7	66	24
Event	Desirable	>7.5	70	<0.3	56	24
	Intermediate	4.5<EI<6	71	<0.6	63	24
	Undesirable	<2.5	142	<0.7	58	24
Person	Desirable	>7	47	1	47	24
	Intermediate	4<PI<5.5	70	<0.6	49	24
	Undesirable	<2	119	<0.6	62	24

Note. Images from the initial stimulus pool (n = 1,245) were rated by an independent sample (n = 41) using a 10-point desirability scale. Stimuli were selected using a two-stage procedure consisting of mean desirability threshold filtering followed by coefficient of variation (CV = SD/mean) filtering. Additional manual screening was performed to remove similar, ambiguous, or potentially uncomfortable images. The final stimulus set consisted of 24 images per desirability level within each semantic category.

S1.2 HUNGER Stimulus Validation and Selection

For the HUNGER experiment, an initial pool of 250 familiar food images was collected to represent a broad range of food incentive values while minimizing differences in visual complexity. All food images were standardized in size and resolution before validation.

Because food preferences are highly individualized, an independent normative rating procedure was conducted using four participants who did not participate in the fMRI experiment (two males and two females) (Furst et al., 1996; Köster, 2009). Participants received the same instructions distinguishing wanting from liking and evaluated their subjective desirability of consuming each food stimulus using the same 10-point desirability scale. These normative ratings were used only for preliminary stimulus balancing and were not used in the neuroimaging analyses.

Food stimuli were categorized according to mean desirability ratings into three groups: desirable food (>6.5), indifferent food (3.5–6.5), and undesirable food (≤3.5). From these categories, final food stimuli were selected by considering desirability

ranking, coefficient of variation, content similarity, ambiguity, and participant comfort. The final food stimulus set included 38 images per desirability category, resulting in 114 food stimuli for the fMRI experiment. The final food stimulus composition and participant-specific food classifications used in subsequent analyses are described in Supplementary Table S7 and Supplementary Figure S3.

The final DESIRE and HUNGER stimulus sets were standardized to 640 × 480 pixels with a resolution of 100 DPI. Images were presented centrally against a neutral background during scanning.

Supplementary Methods S2. Additional Experimental Procedures and GLM Specifications

S2.1 Identification-Based Judgment Task and Exploratory Comparison Analysis

In addition to the desirability judgment runs, the DESIRE experiment included identification-based judgment runs using the same stimulus categories (objects, events, and persons). These runs were designed to provide a non-motivational comparison condition in which participants processed the same stimuli while performing category-specific judgments unrelated to subjective desirability.

For object stimuli, participants were asked to make ownership judgments; for event stimuli, participants evaluated whether they had experienced the depicted event; and for person stimuli, participants performed gender identification judgments. The identification runs used the same event-related structure and stimulus presentation parameters as the desirability judgment runs.

The primary analyses of the present study focused on desirability judgment runs because the central objective was to characterize neural responses associated with motivational desirability and their relationship with physiological food valuation. Nevertheless, to examine whether desirability-related responses could be distinguished from general stimulus-processing demands, additional desirability-versus-identification control analyses were conducted using predefined whole-brain and ROI-level contrasts. These analyses are reported in Supplementary Results SR2, Supplementary Table S8, and Supplementary Figure S4.

S2.2 Modeling of Indifferent Trials and Desirability Contrasts

All behavioral responses collected during the DESIRE and HUNGER experiments were included in the first-level general linear models (GLMs). Specifically, trials were modeled according to the participant's desirability response category, including desirable, indifferent, and undesirable conditions.

For the DESIRE experiment, stimulus category and response category were modeled separately, resulting in nine task regressors for each participant: *Object_desirable, Object_indifferent, Object_undesirable, Event_desirable, Event_indifferent, Event_undesirable, Person_desirable, Person_indifferent, and Person_undesirable* trials. For the HUNGER experiment, desirable, indifferent, and undesirable food trials were modeled separately within each physiological state.

Hunger and Satiety Sessions were modeled independently to account for state-dependent differences in food valuation.

Although indifferent trials were included in the GLM, the primary neuroimaging contrasts focused on the comparison between desirable and undesirable trials. This contrast was selected because the primary objective was to identify neural responses associated with motivational preference at the two ends of the desirability spectrum, rather than to estimate a linear relationship between desirability ratings and neural activity. Additional sensitivity analyses involving indifferent trials were conducted within predefined valuation-related ROIs. Specifically, we examined (1) desirable versus indifferent responses (*Want* > *Neutral*) and (2) indifferent versus undesirable responses (*Neutral* > *Not_Want*) to evaluate whether the primary desirable versus undesirable effects depended on the exclusion of intermediate desirability responses. These analyses are reported in Supplementary Table S8.

S2.3 General Linear Model Specification and Contrast Construction

All neuroimaging analyses were conducted within the GLM framework implemented in SPM8 for the original exploratory whole-brain analyses and SPM12 for subsequent robustness analyses. For each participant, stimulus onset times were convolved with the canonical hemodynamic response function. Six rigid-body motion parameters obtained during realignment (three translations and three rotations) were included as nuisance regressors. The first four functional volumes of each run were discarded before preprocessing to allow magnetization stabilization.

For the DESIRE experiment, individual contrast images were generated for each semantic category by comparing desirable and undesirable trials:

- Object desirability: *Object_desirable* > *Object_undesirable*
- Event desirability: *Event_desirable* > *Event_undesirable*
- Person desirability: *Person_desirable* > *Person_undesirable*

A combined abstract desirability contrast (*Desire_Combined*) was calculated by averaging the three category-specific contrasts: *Desire_Combined* = (OT+ET+PT)/3.

For the HUNGER experiment, categorical food-value responses were assessed using desirable versus undesirable food contrasts across Hunger and Satiety Sessions. Continuous food-value representations were examined using trial-wise parametric modulation models, in which participant-specific food desirability ratings were entered as parametric modulators of stimulus-related BOLD responses. A detailed summary of first-level regressors and second-level contrasts is provided in Supplementary Table S2.

Supplementary Table S2. First-level GLM regressors and second-level contrast definitions

**Panel A. DESIRE first-level regressors**

Regressor	Description
Object_desirable	Object trials rated desirable
Object_indifferent	Object trials rated indifferent
Object_undesirable	Object trials rated undesirable
Event_desirable	Event trials rated desirable
Event_indifferent	Event trials rated indifferent
Event_undesirable	Event trials rated undesirable
Person_desirable	Person trials rated desirable
Person_indifferent	Person trials rated indifferent
Person_undesirable	Person trials rated undesirable
Motion regressors	Six rigid-body motion parameters
Constant	Run-specific constant

Panel B. Second-level contrasts

Contrast	Definition
OT desirability	*Object_desirable* > *Object_undesirable*
ET desirability	*Event_desirable* > *Event_undesirable*
PT desirability	*Person_desirable* > *Person_undesirable*
Desire_Combined	(OT + ET + PT) / 3

Panel C. HUNGER first-level regressors

Session	Regressor
Hunger	*Food_desirable / Food_indifferent / Food_undesirable*
Satiety	*Food_desirable / Food_indifferent / Food_undesirable*
Motion	Six rigid-body motion parameters
Constant	Run-specific constant

Panel D. Food-value models

Model	Definition
Hunger_value_slope	Parametric modulation by Hunger-session desirability ratings
Satiety_value_slope	Parametric modulation by Satiety-session desirability ratings
Hunger > Satiety	*Hunger_value_slope > Satiety_value_slope*
Satiety > Hunger	*Satiety_value_slope > Hunger_value_slope*

Note: First-level regressors and second-level contrasts used for DESIRE and HUNGER neuroimaging analyses. Motion regressors included six rigid-body parameters derived from realignment.

142 **Supplementary Methods S3. ROI Definition and Convergence Analysis**

Procedures

S3.1 Definition of A Priori Valuation-Related Regions of Interest

To examine the robustness of whole-brain findings and to evaluate potential convergence between abstract desirability and food-related valuation, ROI analyses were conducted in three a priori defined valuation-related regions: the anterior cingulate cortex (ACC), medial orbitofrontal cortex (mOFC), and medial prefrontal cortex (mPFC). These regions were selected based on previous neuroimaging studies implicating medial frontal and cingulate systems in subjective value computation, motivational relevance, and integration of internal states with anticipated outcomes (Cardinal et al., 2002; Plassmann et al., 2007; Rangel and Hare, 2010; Rushworth et al., 2011; Bartra et al., 2013; Berridge and Kringelbach, 2015; Rolls, 2023). Anatomical ROI masks were defined using the Automated Anatomical Labeling (AAL) atlas. ROI definitions were specified a priori and were independent of the observed activation patterns. Specifically, the ACC ROI corresponded to the bilateral anterior cingulate cortex mask (AAL Cingulum_Ant), the mOFC ROI corresponded to the bilateral medial orbitofrontal cortex mask (AAL Frontal_Med_Orb), and the mPFC ROI corresponded to the bilateral medial superior frontal gyrus mask (AAL Frontal_Sup_Medial). Because the anatomical mOFC mask extended beyond the functional coverage, ROI analyses were restricted to voxels with valid functional coverage across participants. Effective ROI sizes after coverage restriction were used for all subsequent ROI analyses.

S3.2 ROI Effect Extraction and Statistical Analyses

For each participant, contrast estimates were extracted from the predefined ROIs using the corresponding individual contrast images. ROI effects were summarized using mean parameter estimates across included voxels within each ROI. Abstract desirability analyses included object, event, and person desirability contrasts, as well as the combined abstract desirability contrast (*Desire_Combined*). Food-value analyses included categorical food-value contrasts and continuous food-value models derived from trial-wise parametric modulation analyses.

Group-level ROI effects were assessed using one-sample t-tests. Standardized effect sizes (Cohen's *dz*), 95% confidence intervals, and two-sided *p*-values were calculated. Multiple comparisons across ROI analyses were controlled using Holm correction. Detailed ROI effects for trial-wise food-value analyses are provided in Supplementary Table S3. ROI consistency analyses across object, event, and person desirability domains are reported in Supplementary Table S4.

Supplementary Table S3. ROI effects of trial-wise food-value models

Model	ROI	N	Mean effect	95% CI	*t*	*p*	Holm-corrected *p*	Cohen's *dz*
Hunger_value_slope	mOFC	10	-0.078	[-0.158, 0.002]	-2.203	0.055	0.441	-0.697

Hunger_value_slope	mPFC	10	-0.043	[-0.094, 0.008]	-1.897	0.090	0.632	-0.600
Hunger_value_slope	ACC	10	-0.050	[-0.095, -0.005]	-2.499	0.034	0.305	-0.790
Satiety_value_slope	mOFC	10	-0.081	[-0.181, 0.019]	-1.830	0.101	0.632	-0.579
Satiety_value_slope	mPFC	10	-0.059	[-0.133, 0.015]	-1.806	0.104	0.632	-0.571
Satiety_value_slope	ACC	10	-0.085	[-0.217, 0.048]	-1.446	0.182	0.729	-0.457
Hunger > Satiety_value_slope	mOFC	10	0.003	[-0.104, 0.111]	0.071	0.945	1.000	0.022
Hunger > Satiety_value_slope	mPFC	10	0.016	[-0.064, 0.096]	0.450	0.663	1.000	0.142
Hunger > Satiety_value_slope	ACC	10	0.035	[-0.087, 0.156]	0.649	0.533	1.000	0.205
Satiety > Hunger_value_slope	mOFC	10	-0.003	[-0.111, 0.104]	-0.071	0.945	1.000	-0.022
Satiety > Hunger_value_slope	mPFC	10	-0.016	[-0.096, 0.064]	-0.450	0.663	1.000	-0.142
Satiety > Hunger_value_slope	ACC	10	-0.035	[-0.156, 0.087]	-0.649	0.533	1.000	-0.205

Note: ROI effects were extracted from predefined valuation-related regions (ACC, mOFC, and mPFC) for four trial-wise food-value models: *Hunger_value_slope, Satiety_value_slope, Hunger > Satiety_value_slope,* and *Satiety > Hunger_value_slope*. Mean effects represent mean parameter estimates across voxels within each ROI. Holm correction was applied across the three ROI tests for each food-value model. Cohen's *dz* represents standardized within-sample effect size.

Supplementary Table S4. ROI consistency analyses across object, event, and person desirability domains

**ROI**	**Domain**	**N**	**Mean Estimate**	**95% CI**	***t***	**Cohen's *dz***	**Positive N**	**Negative N**	***P***	**Holm-corrected *P***
ACC	Object desirability	10	0.013	[-0.120, 0.147]	0.228	0.072	4	6	0.754	1.000
	Event	10	-0.009	[-0.099,	-0.217	-0.069	5	5	1.000	1.000

	desirability			0.082]						
	Person desirability	10	-0.093	[-0.258, 0.072]	-1.278	-0.404	2	8	0.109	1.000
mOFC	Object desirability	10	0.034	[-0.094, 0.161]	0.600	0.190	8	2	0.109	1.000
	Event desirability	10	0.027	[-0.068, 0.122]	0.643	0.203	7	3	0.344	1.000
	Person desirability	10	-0.118	[-0.222, -0.013]	-2.552	-0.807	2	8	0.109	0.249
mPFC	Object desirability	10	-0.060	[-0.260, 0.139]	-0.683	-0.216	4	6	0.754	1.000
	Event desirability	10	0.037	[-0.037, 0.111]	1.136	0.359	7	3	0.344	1.000
	Person desirability	10	-0.196	[-0.345, -0.047]	-2.981	-0.943	1	9	0.021	0.139

Note: ROI effects were extracted for object, event, and person desirability contrasts within predefined valuation-related regions (ACC, mOFC, and mPFC). Positive N and Negative N indicate the number of participants showing positive and negative parameter estimates, respectively. Multiple comparisons across ROI analyses were controlled using Holm correction. Nominal uncorrected effects were interpreted cautiously and were not considered significant after correction for multiple comparisons.

S3.3 Cross-Domain Convergence Analyses

To examine whether abstract desirability and food incentive valuation showed convergent neural responses within predefined valuation-related regions, complementary ROI-based convergence analyses were conducted. The primary convergence analysis compared abstract desirability responses (*Desire_Combined*) with continuous food-value coding during Hunger (*Hunger_value_slope*) within the ACC, mOFC, and mPFC. Secondary analyses examined convergence between *Desire_Combined* and hunger-related modulation of food-value coding (*Hunger_value_slope* > *Satiety_value_slope*), and categorical food-value responses (*Desirable* > *Undesirable*).

Cross-domain correspondence was evaluated using participant-level correlations between abstract desirability and food-value effects, together with sign-consistency analyses assessing whether participants showed concordant positive effects across domains. Detailed ROI-based convergence results are provided in Supplementary Table S5. ROI-level distributions associated with abstract desirability and food-value analyses are illustrated in Supplementary Figure S2.

Supplementary Table S5. ROI-based cross-domain convergence analyses between abstract desirability and food valuation

Panel A. ***Desire_Combined vs Hunger_value_slope***

ROI	Component	N	Mean effect	95% CI	*t*	*p*	Holm-corrected *p*	Cohen's *dz*	Positive N	Negative N
ACC	*Desire_Combined*	10	-0.030	[-0.112, 0.053]	-0.812	0.438	0.875	-0.257	4	6
	Hunger_Value_Slope	10	-0.050	[-0.095, -0.005]	-2.499	0.034	0.204	-0.790	3	7
mOFC	*Desire_Combined*	10	-0.019	[-0.074, 0.036]	-0.785	0.452	0.875	-0.248	3	7
	Hunger_Value_Slope	10	-0.078	[-0.158, 0.002]	-2.212	0.054	0.271	-0.699	2	8
mPFC	*Desire_Combined*	10	-0.073	[-0.175, 0.029]	-1.626	0.138	0.415	-0.514	3	7
	Hunger_Value_Slope	10	-0.042	[-0.093, 0.009]	-1.868	0.095	0.378	-0.591	3	7

Panel B. ***Desire_Combined vs Hunger_Minus_Satiety_Slope***

ROI	Component	N	Mean effect	95% CI	*t*	*p*	Holm-corrected *p*	Cohen's *dz*	Positive N	Negative N
ACC	*Desire_Combined*	10	-0.030	[-0.112, 0.053]	-0.812	0.438	1.000	-0.257	4	6
	Hunger_Minus_Satiety_Slope	10	0.035	[-0.087, 0.156]	0.649	0.533	1.000	0.205	7	3
mOFC	*Desire_Combined*	10	-0.019	[-0.074, 0.036]	-0.785	0.452	1.000	-0.248	3	7
	Hunger_Minus_Satiety_Slope	10	0.003	[-0.104, 0.111]	0.069	0.947	1.000	0.022	6	4
mPFC	*Desire_Combined*	10	-0.073	[-0.175, 0.029]	-1.626	0.138	0.830	-0.514	3	7
	Hunger_Minus_Satiety_Slope	10	0.017	[-0.065, 0.099]	0.467	0.652	1.000	0.148	7	3

Panel C. ***Desire_Combined vs Categorical_Food***

ROI	Component	N	Mean effect	95% CI	*t*	*p*	Holm-corrected	Cohen's *dz*	Positive	Negative

							p		N	N
ACC	*Desire_Combined*	8	-0.039	[-0.111, 0.034]	-1.269	0.245	1	-0.449	3	5
	Categorical_Food	8	-0.031	[-0.192, 0.129]	-0.459	0.66	1	-0.162	3	5
mOFC	*Desire_Combined*	8	-0.027	[-0.093, 0.040]	-0.94	0.378	1	-0.332	2	6
	Categorical_Food	8	-0.13	[-0.358, 0.098]	-1.349	0.219	1	-0.477	2	6
mPFC	*Desire_Combined*	8	-0.055	[-0.145, 0.035]	-1.439	0.193	1	-0.509	2	6
	Categorical_Food	8	-0.041	[-0.170, 0.088]	-0.756	0.474	1	-0.267	3	5

Note: *Desire_Combined* represents the averaged desirability contrast across object, event, and person domains. *Hunger_value_slope* represents continuous food-value coding during the Hunger Session. *Hunger_Minus_Satiety_Slope* represents the difference between food-value coding during Hunger and Satiety Sessions. *Categorical_Food* represents the *Desirable > Undesirable* food contrast. ROI effects were extracted from predefined valuation-related regions (ACC, mOFC, and mPFC). Pearson correlations and sign-consistency analyses were used to evaluate cross-domain correspondence where applicable. Multiple comparisons were corrected using Holm correction. The reduced sample size for *Categorical_Food* analyses reflects participants who lacked sufficient trials for this contrast.

[Insert Supplementary Figure S2 here.]

Supplementary Methods S4. Spatial Overlap and Permutation-Based Robustness Procedures

S4.1 Whole-Brain Spatial Overlap Analyses

To assess whether abstract desirability and food-value representations showed spatial correspondence beyond ROI-based analyses, whole-brain spatial overlap analyses were conducted between thresholded statistical maps. Spatial correspondence was quantified using Dice and Jaccard similarity coefficients, which have been previously applied to evaluate spatial overlap and reproducibility between neuroimaging activation maps (Rombouts et al., 1998). For two binary activation maps (A and B), Dice overlap was calculated as: $Dice = 2|A \cap B| / (|A| + |B|)$, and Jaccard similarity was calculated as: $Jaccard = |A \cap B| / |A \cup B|$.

Overlap analyses were performed between the combined abstract desirability contrast (*Desire_Combined*) and three food-value-related contrasts: (1) continuous food-value coding during Hunger (*Hunger_value_slope*); (2) hunger-related modulation of food-value coding (*Hunger_value_slope > Satiety_value_slope*); and (3) categorical food-value responses (*Categorical_Food: Desirable > Undesirable*).

Binary activation maps were generated using the same exploratory thresholding schemes applied in the corresponding whole-brain analyses. Resulting overlap statistics, including voxel counts, Dice coefficients, and Jaccard coefficients, are summarized in Supplementary Table S9.

S4.2 Permutation-Based Robustness Analyses

To determine whether observed cross-domain correspondence exceeded chance expectations, participant-level permutation analyses were conducted using two complementary approaches. First, ROI-level correlation analyses were evaluated by comparing observed Pearson correlations between abstract desirability and food-value effects with null distributions generated by randomly shuffling participant labels. Empirical permutation *p*-values were calculated as the proportion of permuted correlations exceeding the observed correlation magnitude.

Second, sign-consistency analyses assessed whether the number of participants showing positive effects across both abstract desirability and food-value measures exceeded chance expectations. The observed number of participants with concordant positive effects was compared with permutation-derived null distributions.

Permutation results, including observed statistics, null distributions, and empirical permutation *p*-values, are reported in Supplementary Table S10.

Supplementary Results SR1. Behavioral validation of physiological manipulation and food classification

To further characterize the physiological-state manipulation, participant-level fullness ratings collected during the DESIRE baseline, Hunger, and Satiety Sessions are provided in Supplementary Table S6. These individual ratings complement the group-level analyses reported in the main manuscript and demonstrate variability across participants while confirming successful separation of baseline, Hunger, and Satiety states.

Supplementary Table S6. Participant-level fullness ratings across experimental sessions

Subject	DESIRE baseline	Hunger Session before fasting	Hunger Session before scanning	Satiety Session before scanning
101	6	8	2	9
102	5	8	3	10
103	8	8	2	9
104	5	10	2	10
105	5	7	3	9
106	6	10	3	9
107	7	10	2	10
108	8	9	2	10
109	7	8	3	8.5

110	6	10	3	10

Note: Fullness ratings were assessed using a 10-point visual analogue scale (VAS; 1 = extremely hungry, 10 = extremely full). DESIRE baseline ratings were collected before scanning under the non-deprived condition. Hunger-before-fasting ratings were obtained before the fasting manipulation, whereas Hunger and Satiety ratings were collected immediately before fMRI acquisition.

Participant-specific food classifications used in subsequent categorical analyses were examined across all paired Hunger and Satiety observations. Hunger Foods represented food items whose desirability increased during Hunger, whereas Neutral Foods represented items with unchanged desirability across physiological states. Irregular Foods reflected opposite-direction transitions or invalid paired responses. Participant-level classification results are summarized in Supplementary Table S7 and illustrated in Supplementary Figure S3.

Supplementary Table S7. Participant-specific food classification across Hunger and Satiety Sessions

Subject	Hunger Food	Neutral Food	Irregular Food	Missing/Invalid Pairs	Valid Paired Trials
101	72	38	4	0	114
102	75	38	1	0	114
103	89	23	1	0	113
104	52	62	0	0	114
105	88	26	0	0	114
106	31	68	8	0	107
107	59	54	0	0	113
108	79	35	0	0	114
109	66	40	8	0	114
110	48	61	5	0	114

Note: Food classifications were based on within-participant changes in desirability ratings between Hunger and Satiety Sessions. Hunger Foods indicated increased desirability during Hunger, Neutral Foods indicated unchanged desirability, and Irregular Foods represented opposite-direction transitions or invalid paired responses. Irregular Food trials were excluded from the primary Hunger Food versus Neutral Food contrast.

[Insert Supplementary Figure S3 here.]

Supplementary Results SR2. Identification-Related Exploratory Analyses

To examine whether desirability-related responses could be distinguished from responses associated with broader stimulus-processing demands, exploratory comparisons between desirability and identification judgment runs were conducted.

Three predefined contrasts were examined. Contrast 19 (*Desire_runs* >

Identification_runs) assessed the overall difference between desirability and identification judgments across corresponding runs. Contrast 49 (*Desire_Positive > Identification_Positive*) assessed positive-response differences between desirability and identification conditions. Contrast 51 (*Desire_Valued_Diff > Identification_Response_Diff*) evaluated a difference-of-differences effect comparing desirability-related value differences with identification-related response differences.

Contrast 19 and Contrast 49 did not reveal suprathreshold clusters at the exploratory threshold of $p < 0.001$ (uncorrected) with a cluster extent threshold of $k \geq 20$ voxels. Contrast 51 revealed one exploratory suprathreshold cluster under the same threshold; however, this cluster did not survive cluster-level family-wise error correction (cluster-level $p = 0.954$). These analyses are illustrated in Supplementary Figure S4.

[Insert Supplementary Figure S4 here.]

ROI-based analyses of identification-related contrasts within the predefined valuation-related regions (ACC, mOFC, and mPFC) did not reveal robust effects after correction for multiple comparisons (Supplementary Table S8-Panel B). Because the identification contrasts involved heterogeneous cognitive operations, including ownership judgments, event experience judgments, and gender identification, these analyses were interpreted as control analyses rather than as a pure non-motivational baseline. Accordingly, the primary findings are interpreted as reflecting desirability-related neural responses rather than responses uniquely isolated from all non-motivational processing demands.

Supplementary Results SR3. Indifferent-Condition Sensitivity Analyses

To examine whether the interpretation of the primary desirability effects depended on the exclusion of indifferent trials, additional ROI-based sensitivity analyses were conducted using alternative desirability contrasts.

Two contrasts were examined: (1) *Want > Neutral*, assessing whether desirable responses differed from intermediate desirability responses; and (2) *Neutral > Not_Want*, assessing whether indifferent responses remained distinguishable from undesirable responses.

Across the predefined valuation-related ROIs (ACC, mOFC, and mPFC), both sensitivity contrasts showed small and inconsistent effects. *Want > Neutral* responses showed small positive effects in the ACC, mOFC, and mPFC (0.038, 0.043, and 0.016, respectively), whereas *Neutral > Not_Want* responses showed negative effects across these regions (-0.067, -0.062, and -0.089, respectively).

These findings indicate that the primary desirable-versus-undesirable effects were not explained solely by the treatment of indifferent trials, as alternative contrasts involving intermediate desirability responses did not reveal a consistent pattern within the predefined valuation-related regions. Complete ROI-based sensitivity results are provided in Supplementary Table S8-Panel A.

Supplementary Table S8. Additional sensitivity analyses involving indifferent trials and identification-related exploratory comparisons

A. Indifferent-condition sensitivity analyses

Contrast	ACC	mOFC	mPFC
Want > Indifferent	0.038	0.043	0.016
Indifferent > Not_Want	-0.067	-0.062	-0.089

B. Identification-related exploratory comparisons

ContrastName	ROI	N	Mean	95% CI	*t*	*p*	Holm -corrected *p*	Cohen's *dz*
19_Desire_runs > Identification_runs	mOFC	10	0.052	[-0.217, 0.321]	0.436	0.673	1	0.138
19_Desire_runs > Identification_runs	mPFC	10	0.174	[-0.078, 0.426]	1.565	0.152	1	0.495
19_Desire_runs > Identification_runs	ACC	10	0.048	[-0.291, 0.386]	0.319	0.757	1	0.101
49_Desire_Positive > Identification_Positive	mOFC	10	0.128	[-0.120, 0.377]	1.171	0.272	1	0.370
49_Desire_Positive > Identification_Positive	mPFC	10	0.200	[-0.055, 0.456]	1.773	0.110	0.991	0.561
49_Desire_Positive > Identification_Positive	ACC	10	0.077	[-0.266, 0.420]	0.507	0.625	1	0.160
51_Desire_Value_Diff > Identification_Response_Diff	mOFC	10	0.096	[-0.034, 0.227]	1.676	0.128	1	0.530
51_Desire_Value_Diff > Identification_Response_Diff	mPFC	10	0.000	[-0.089, 0.090]	0.009	0.993	1	0.003
51_Desire_Value_Diff > Identification_Response_Diff	ACC	10	0.031	[-0.092, 0.154]	0.569	0.583	1	0.180

Note: Panel A reports ROI-based sensitivity analyses using alternative desirability contrasts (*Want* > *Indifferent* and *Indifferent* > *Not_Want*) to evaluate whether the primary *Want* > *Not_Want* effects depended on the exclusion of intermediate desirability responses. Panel B reports ROI-based exploratory comparisons between desirability and identification-related responses for three predefined contrasts: Contrast 19 (*Desire_runs* > *Identification_runs)*, Contrast 49 (*Desire_Positive* > *Identification_Positive*), and Contrast 51 (*Desire_Valued_Diff* > *Identification_Response_Diff*). Identification-related contrasts were interpreted cautiously because they combine heterogeneous cognitive operations rather than representing a pure non-motivational control condition. Holm correction was applied to ROI-level analyses.

Supplementary Results SR4. Whole-brain overlap and permutation-based robustness analyses

To further evaluate spatial correspondence between abstract desirability and food-value responses, whole-brain overlap analyses were conducted between thresholded statistical maps. Overlap was assessed using Dice and Jaccard coefficients across three comparisons: (1) *Desire_Combined* versus *Hunger_value_slope*; (2) *Desire_Combined* versus hunger-related value modulation (*Hunger_value_slope* > *Satiety_value_slope*); and (3) *Desire_Combined* versus *Categorical_Food* value responses.

Across all comparisons and exploratory thresholding schemes, no suprathreshold voxel overlap was observed between abstract desirability and food-value maps. Detailed overlap statistics, including overlap voxel counts, Dice coefficients, and Jaccard coefficients, are reported in Supplementary Table S9.

Supplementary Table S9. Whole-brain spatial overlap analyses between abstract desirability and food-value maps

Comparison	Threshold	Map A	Map B	Voxel Count A	Voxel Count B	Overlap Voxels	Dice	Jaccard
Desire_vs_Hunger_Slope	p001_k0	*Desire_Combined*	*Hunger_Value_Slope*	64	10	0	0	0
Desire_vs_Hunger_Minus_Satiety	p001_k0	*Desire_Combined*	*Hunger_Minus_Satiety*	64	2	0	0	0
Desire_vs_Categorical_Food	p001_k0	*Desire_Combined*	*Categorical_Food*	64	1	0	0	0
Desire_vs_Hunger_Slope	p005_k20	*Desire_Combined*	*Hunger_Value_Slope*	237	21	0	0	0
Desire_vs_Hunger_Minus_Satiety	p005_k20	*Desire_Combined*	*Hunger_Minus_Satiety*	237	0	0	0	0
Desire_vs_Categorical_Food	p005_k20	*Desire_Combined*	*Categorical_Food*	237	0	0	0	0

Note: Spatial overlap was quantified between thresholded whole-brain statistical maps

using Dice and Jaccard coefficients. Map A represented the *Desire_Combined* contrast, whereas Map B represented food-value-related contrasts. No suprathreshold voxel overlap was observed across comparisons. p001_k0 and p001_k20 denote voxel-wise thresholds of $p < 0.001$ (uncorrected) with cluster-extent thresholds of 0 and 20 contiguous voxels, respectively.

Permutation-based analyses further evaluated whether observed participant-level correspondence exceeded chance expectations. Correlation-based permutation tests comparing ROI-level cross-domain associations against shuffled null distributions did not reveal significant correspondence. Similarly, sign-consistency permutation tests indicated that the number of participants showing concordant positive effects across abstract desirability and food-value measures did not exceed chance expectations. Permutation results are summarized in Supplementary Table S10.

Supplementary Table S10. Permutation-based robustness analyses of cross-domain convergence

Panel A. ROI correlation permutation tests

**Comparison**	**ROI**	**N**	***r***	***p***	**Permutation *p***	**Null Mean *r***	**Null SD**
Desire_vs_Hunger_Value_Slope	ACC	10	0.199	0.581	0.577	0.001	0.332
Desire_vs_Hunger_Value_Slope	mOFC	10	0.605	0.064	0.062	-0.001	0.333
Desire_vs_Hunger_Value_Slope	mPFC	10	0.574	0.083	0.083	0.005	0.334
Desire_vs_Hunger_Minus_Satiety	ACC	10	-0.192	0.595	0.605	-0.002	0.333
Desire_vs_Hunger_Minus_Satiety	mOFC	10	0.319	0.369	0.377	0.001	0.338
Desire_vs_Hunger_Minus_Satiety	mPFC	10	-0.119	0.744	0.746	0.004	0.337
Desire_vs_Categorical_Food	ACC	8	-0.682	0.062	0.053	0.002	0.375
Desire_vs_Categorical_Food	mOFC	8	0.065	0.879	0.887	0.002	0.381
Desire_vs_Categorical_Food	mPFC	8	0.081	0.848	0.846	0.003	0.377

Panel B. Both-positive sign consistency permutation tests

**Comparison**	**ROI**	**Observed Both-positive N**	**Permutation *p***	**Null Mean N**	**Null SD**

Desire_vs_Hunger_Value_Slope	ACC	2	0.338	1.203	0.748
Desire_vs_Hunger_Value_Slope	mOFC	2	0.066	0.599	0.610
Desire_vs_Hunger_Value_Slope	mPFC	1	0.702	0.892	0.703
Desire_vs_Hunger_Minus_Satiety	ACC	3	0.660	2.792	0.757
Desire_vs_Hunger_Minus_Satiety	mOFC	3	0.166	1.800	0.749
Desire_vs_Hunger_Minus_Satiety	mPFC	3	0.299	2.107	0.707
Desire_vs_Categorical_Food	ACC	1	0.825	1.129	0.704
Desire_vs_Categorical_Food	mOFC	0	1.000	0.495	0.561
Desire_vs_Categorical_Food	mPFC	1	0.639	0.746	0.635

Note: Permutation analyses evaluated whether observed participant-level correspondence exceeded chance expectations. Pearson correlations were compared against null distributions generated by random permutation of participant labels. Sign-consistency tests evaluated whether the number of participants showing positive effects across domains exceeded permutation-derived expectations.

References:

Bartra, O., McGuire, J. T., and Kable, J. W. (2013). The valuation system: A coordinate-based meta-analysis of BOLD fMRI experiments examining neural correlates of subjective value. *NeuroImage* 76, 412–427. doi: 10.1016/j.neuroimage.2013.02.063

Berridge, K. C. (2009). Wanting and Liking: Observations from the Neuroscience and Psychology Laboratory. *Inq. Oslo Nor.* 52, 378. doi: 10.1080/00201740903087359

Berridge, K. C., and Kringelbach, M. L. (2015). Pleasure Systems in the Brain. *Neuron* 86, 646–664. doi: 10.1016/j.neuron.2015.02.018

Berridge, K. C., and Robinson, T. E. (2016). Liking, Wanting and the Incentive-Sensitization Theory of Addiction. *Am. Psychol.* 71, 670–679. doi: 10.1037/amp0000059

Bosulu, J., Allaire, M., Tremblay-Grénier, L., Luo, Y., Eickhoff, S., and Hétu, S. (2022). “Wanting” versus “needing” related value: An fMRI meta-analysis. *Brain Behav.* 12, e32713. doi: 10.1002/brb3.2713

Cardinal, R. N., Parkinson, J. A., Hall, J., and Everitt, B. J. (2002). Emotion and motivation: the role of the amygdala, ventral striatum, and prefrontal cortex. *Neurosci. Biobehav. Rev.* 26, 321–352. doi: 10.1016/S0149-7634(02)00007-6

Furst, T., Connors, M., Bisogni, C. A., Sobal, J., and Falk, L. W. (1996). Food Choice: A Conceptual Model of the Process. *Appetite* 26, 247–266. doi: 10.1006/appe.1996.0019

Grabenhorst, F., and Rolls, E. T. (2011). Value, pleasure and choice in the ventral prefrontal cortex. *Trends Cogn. Sci.* 15, 56–67. doi: 10.1016/j.tics.2010.12.004

Köster, E. P. (2009). Diversity in the determinants of food choice: A psychological perspective. *Food Qual. Prefer.* 20, 70–82. doi: 10.1016/j.foodqual.2007.11.002

Morales, I., and Berridge, K. C. (2020). ‘Liking’ and ‘wanting’ in eating and food reward: Brain mechanisms and clinical implications. *Physiol. Behav.* 227, 113152. doi: 10.1016/j.physbeh.2020.113152

Nguyen, D., Naffziger, E. E., and Berridge, K. C. (2021). Positive Affect: Nature and brain bases of liking and wanting. *Curr. Opin. Behav. Sci.* 39, 72–78. doi: 10.1016/j.cobeha.2021.02.013

Plassmann, H., O’Doherty, J., and Rangel, A. (2007). Orbitofrontal Cortex Encodes Willingness to Pay in Everyday Economic Transactions. *J. Neurosci.* 27, 9984–9988. doi: 10.1523/JNEUROSCI.2131-07.2007

Rangel, A., and Hare, T. (2010). Neural computations associated with goal-directed choice. *Curr. Opin. Neurobiol.* 20, 262–270. doi: 10.1016/j.conb.2010.03.001

Rolls, E. T. (2023). Emotion, motivation, decision-making, the orbitofrontal cortex, anterior cingulate cortex, and the amygdala. *Brain Struct. Funct.* 228, 1201–1257. doi: 10.1007/s00429-023-02644-9

Rombouts, S. A. R. B., Barkhof, F., Hoogenraad, F. G. C., Sprenger, M., and Scheltens, P. (1998). Within-Subject Reproducibility of Visual Activation Patterns With Functional Magnetic Resonance Imaging Using Multislice Echo Planar Imaging. *Magn. Reson. Imaging* 16, 105–113. doi: 10.1016/S0730-725X(97)00253-1

Rushworth, M. F. S., Noonan, M. P., Boorman, E. D., Walton, M. E., and Behrens, T. E. (2011). Frontal Cortex and Reward-Guided Learning and Decision-Making. *Neuron* 70, 1054–1069. doi: 10.1016/j.neuron.2011.05.014

Supplementary T1. Behavioral validation procedure for DESIRE stimulus selection. Independent behavioral validation procedure for selecting DESIRE stimuli. Participants received instructions distinguishing motivational wanting from hedonic liking and rated object, event, and person images using a 10-point desirability scale. Ratings were used for two-stage stimulus selection based on mean desirability thresholds and coefficient of variation criteria.

Supplementary Figure S2. ROI-level effect distributions and cross-domain correspondence analyses. ROI-level analyses across predefined valuation-related regions. (A) ROI effects associated with object, event, and person desirability judgments are shown for the ACC, mOFC, and mPFC. (B) Continuous food-value coding effects are shown across Hunger and Satiety Sessions based on trial-wise parametric modulation analyses. (C) Participant-level correspondence between abstract desirability (*Desire_Combined*) and continuous food-value coding during Hunger (*Hunger_value_slope*) is illustrated for each ROI. Pearson correlation coefficients are shown for visualization purposes; none of the cross-domain associations survived permutation-based correction.

Supplementary Figure S3. Participant-level classification of food stimuli across Hunger and Satiety Sessions. Participant-specific classification of food stimuli based on changes in desirability ratings between Hunger and Satiety Sessions. Panel A shows trial-level classification for each participant. Blue indicates Hunger Food trials, gray indicates Neutral Food trials, red indicates Irregular Food trials, and white indicates missing or invalid paired trials. Panel B summarizes the number of trials assigned to each category across participants. Hunger Foods were defined as food items rated as more desirable during Hunger than Satiety, Neutral Foods were defined as items showing unchanged desirability, and Irregular Foods represented opposite-direction transitions or invalid paired responses. Irregular Food trials were excluded from the primary Hunger Food versus Neutral Food contrast. Classifications were performed within participants to account for individual differences in food preference.

Supplementary Figure S4. Exploratory comparisons between desirability and identification-related responses. (A) Exploratory comparisons between desirability judgment runs and identification-based judgment runs. Contrast 19 represented the overall difference between desirability and identification tasks across corresponding runs and did not reveal suprathreshold clusters. Contrast 49 represented a historical positive-response contrast combining desirability and identification responses with different task meanings and did not reveal suprathreshold clusters. Contrast 51 represented a historical difference-of-differences contrast comparing desirability value differences with heterogeneous identification response differences. (B) One exploratory cluster was identified at $p < 0.001$ uncorrected with $k \geq 20$ voxels. Orthogonal slices showing the cluster identified in Contrast 51. The peak voxel was located at MNI coordinate [-9, -64, 25] with $T = 6.53$. The identified cluster did not survive cluster-level FWE correction (cluster-level $p = 0.954$). These analyses were interpreted cautiously because the identification contrasts combine different cognitive

operations rather than isolating a pure non-motivational control process.
